# The effects of hesperidin supplementation on cardiovascular risk factors in adults: a systematic review and dose–response meta-analysis

**DOI:** 10.3389/fnut.2023.1177708

**Published:** 2023-07-12

**Authors:** Atie Sadat Khorasanian, Sahand Tehrani Fateh, Fatemeh Gholami, Niloufar Rasaei, Hadis Gerami, Sayyed Saeid Khayyatzadeh, Farideh Shiraseb, Omid Asbaghi

**Affiliations:** ^1^Department of Nutrition, School of Public Health, Iran University of Medical Sciences, Tehran, Iran; ^2^School of Medicine, Tehran University of Medical Sciences, Tehran, Iran; ^3^Department of Community Nutrition, School of Nutritional Sciences and Dietetics, Tehran University of Medical Sciences (TUMS), Tehran, Iran; ^4^Nutrition and Food Security Research Center, Shahid Sadoughi University of Medical Sciences, Yazd, Iran; ^5^Endocrinology and Metabolism Research Center, Endocrinology and Metabolism Clinical Sciences Institute, Tehran University of Medical Sciences, Tehran, Iran; ^6^Department of Nutrition, Faculty of Health, Shahid Sadoughi University of Medical Sciences, Yazd, Iran; ^7^Cancer Research Center, Shahid Beheshti University of Medical Sciences, Tehran, Iran; ^8^Student Research Committee, Shahid Beheshti University of Medical Sciences, Tehran, Iran

**Keywords:** hesperidin, lipid profile, blood pressure, body mass index, meta-analysis, fasting blood glucose, adults

## Abstract

Hesperidin is a naturally occurring bioactive compound that may have an impact on cardiovascular disease risks, but the evidence is not conclusive. To investigate further, this study aimed to explore the effects of hesperidin supplementation on cardiovascular risk factors in adults. A comprehensive search was conducted up to August 2022 using relevant keywords in databases such as Scopus, PubMed, Embase, Cochrane Library, and ISI Web of Science for all randomized controlled trials (RCTs). The results showed that hesperidin supplementation had a significant effect on reducing serum triglyceride (TG), total cholesterol (TC), low-density cholesterol (LDL), tumor necrosis factor-alpha (TNF-α), and systolic blood pressure (SBP), whereas weight was increased. However, no significant effect was observed on high-density cholesterol (HDL), waist circumference (WC), fasting blood glucose (FBG), insulin, homeostatic model assessment for insulin resistance (HOMA-IR), C-reactive protein (CRP), interleukin-6 (IL-6), body mass index (BMI), and diastolic blood pressure (DBP). The study also found that an effective dosage of hesperidin supplementation was around 1,000 mg/d, and a more effective duration of supplementation was more than eight weeks to decrease insulin levels. Furthermore, the duration of intervention of more than six weeks was effective in decreasing FBG levels.

## 1. Introduction

Cardiovascular diseases (CVDs) are the leading cause of death globally, with consistently increasing morbidity and mortality rates (1). Approximately 31% of global deaths are attributed to CVDs (2, 3). Poor health habits and various diseases, such as dyslipidemia, hyperglycemia, hypertension, and inflammatory disease, can increase CVD mortality related to acute myocardial infarction and stroke, both synergistically and separately (4–7). In addition to pharmacotherapy and lifestyle modifications such as dietary interventions and weight loss, nutritional interventions can also help manage CVD risk factors and metabolic disorders (8–11). CVDs are largely caused by inappropriate diet and lifestyle, so improving dietary habits and making them more accessible to the general population is the primary strategy for preventing the onset of CVDs and CVD risk factors (12–14). Some CVD risk factors, such as hypertension, diabetes, and hypercholesterolemia, can be improved by using alternative treatments such as natural-based products, given the benefits associated with diet in CVD development (13, 15). Using alternative therapies, including bioactive phytoconstituents in traditional medicinal plants, could help decrease and manage CVD risk factors and metabolic disorders (16–19).

Polyphenols, a large group of natural-based bioactive compounds, consist of three main classes, including flavonoids and non-flavonoids (20, 21). Many vegetables and fruits contain bioactive metabolites, such as the flavonoid family, which includes over 15,000 molecules (22–24). Citrus fruits are particularly high in flavonoids, with one of the most well-known being hesperidin, which exists in two isomeric forms, 2S- and 2R- (23, 25). Oranges and their juice are abundant sources of hesperidin and naringin, with over 90% of sweet orange flavonoids deriving from these compounds (26). Hesperidin is transformed into hesperetin (aglycon) by the bacterial flora of the intestine, which is the main metabolite of this flavonoid (13). Hesperidin has anti-inflammatory properties and has positive effects on various diseases, including insulin resistance, non-alcoholic fatty liver disease, metabolic syndrome, and CVDs (27–29). However, factors such as bacterial flora transformation, bioavailability, and absorption can affect the performance of flavonoids, including hesperidin (30).

Research has shown that hesperidin treatment can affect CVD risk factors, such as insulin resistance (31), hyperglycemia (32), blood pressure and vascular endothelial function (33), hypercholesterolemia (34), and inflammation (35) in both *in vitro* and *in vivo* studies. While there is great potential to leverage bioactive components of plants for the discovery and development of modern therapies, the results from randomized controlled trials (RCTs) examining the effect of hesperidin supplementation on the constellation of risk factors for CVD risk factors (total cholesterol [TC], triglyceride [TG], low-density lipoprotein [LDL], high-density lipoprotein [HDL], fasting blood glucose [FBG], insulin, homeostatic model assessment for insulin resistance [HOMA-IR], systolic blood pressure consisting of systolic blood pressure [SBP] and diastolic blood pressure [DBP], inflammatory markers such as C-reactive protein [CRP], interleukin 6 [IL-6], tumor necrosis factor [TNF-α], and even anthropometric indices such as weight, body mass index [BMI] and waist circumference [WC]) are inconclusive due to high variability between human trials. Also, the addition of new articles about CVD risk factors, as well as a complete assessment of all CVD risk factors, is the reason for this systematic review. Therefore, this study aimed to evaluate the effect of hesperidin supplementation on cardiovascular disease risk factors in adults.

## 2. Methods

PRISMA declaration was used in this study for reporting preferred reporting items for systematic reviews and meta-analyses (36).

### 2.1. Search strategy

PubMed/Medline, Scopus, Web of Science, EMBASE, and the Cochrane databases, as well as Google Scholar, were searched to identify available RCT on hesperidin supplementation and CVD risk factors published up to August 2022. Additionally, research bibliographies were reviewed to identify any potential missing studies. Neither the time nor the language of the publications were restricted. We used the PICO (Participant, Intervention, Comparison/Control, Outcome) search framework to search for items related to hesperidin supplementation and CVD risk factors and to search for items related to hesperidin supplementation and all the outcomes. We used relevant formatting for each folder. We used a combination of MeSH terms and keywords as the first step of the data collection process, to ensure that no studies were missed. The reference lists for all related studies were manually searched using the following keywords: (“hesperidin” OR “hesperitin” OR “citrus flavonoid” OR “orange juice” OR “citrus flavanones” OR “orange polyphenols”) AND (Intervention OR “Intervention Study” OR “Intervention Studies” OR Randomized OR Random OR Randomly OR Placebo OR “Clinical Trial” OR Trial OR Trials OR “Randomized Clinical Trial” OR RCT OR blinded OR “double blind” OR “double blinded” “Controlled Trial” “Randomized Controlled Trial” OR “Controlled Clinical Trial” OR “Pragmatic Clinical Trial” OR “Cross-Over Studies” OR “Cross-Over” OR “Cross-Over Study” OR Parallel OR “Parallel Study” OR “Parallel trial”).

### 2.2. Study selection

Research studies were included if they met the following criteria: (1) RCTs (parallel or cross-over); (2) oral intake of hesperidin; (3) assessed the effects of hesperidin supplementation on CVD risk factors; (4) RCTs with two or more eligible arms were considered as separate studies; (5) study participants were adults (≥18 years old); (6) provided means and standard deviations (SDs) for hesperidin, provided means and SDs for CVD risk factors or any other effect sizes from which the calculation of mean and SD was possible. No language restrictions were imposed on the searches, and only human studies were included. Two authors (ASK and OA) independently assessed the validity of the qualifying studies by assessing their titles and abstracts, extracting results, and investigating the validity of the included publications. The following studies were excluded: animal studies, reviews, and studies conducted *in vitro*. Additionally, we excluded gray literature, conference abstracts, editorial papers, books, and RCTs without a placebo or control group. Similarly, studies with the combination of hesperidin with vitamins or minerals, without a concurrent placebo-controlled group, or that reported mean CVDs to risk factors [TC (mg/dl), TG (mg/dl), LDL (mg/dl), HDL (mg/dl), FBG (mg/dl), insulin (μU/mL), HOMA-IR, SBP (mmHg), DBP (mm Hg), baseline BMI (kg/m^2^), weight (kg), WC (cm), CRP (mg/l), IL-6 (pg/mL) and TNF-α (pg/mL)] as the only outcome were excluded.

### 2.3. Data extraction

The authors ASK and OA independently re-checked all eligible RCTs and extracted the following information. Several data were extracted for further analysis, including the first author’s name, country, year of publication, type of clinical study, characteristics of participants (age, BMI, and sex), randomization, blinding, sample size, dose, and forms of supplemented hesperidin, duration, and related information. We collected the mean and SD for CVD risk factors at the start and end of each intervention (for both parallel and cross-over trials). Whenever no such data were available, the mean difference was calculated by subtracting the mean value of the baseline from that of the endpoint. If the hesperidin doses were reported in g/day, we converted them to mg/day.

### 2.4. Quality assessment

Authors ASK and OA separately assessed the quality of the work, with any differences settled by discussion. We used the Cochrane Collaboration tool to assess the quality of the studies (37). A series of seven items, including randomization sequence generation, allocation concealment, participant and staff blinding, outcome assessor blinding, poor outcome data, selective reporting, and other biases were identified in all studies included in the review. Table 1 summarizes the results of the analysis. The studies were divided into three groups: high risk of bias group (general high risk >2 high risks), low risk of bias group (general low risk <2 high risks), and moderate risk of bias group (general moderate risk = 2 high risks).

**Table 1:** GRADE profile of hesperidin supplementation on cardiovascular risk factors.

Quality assessment						Summary of findings		Quality of evidence
Outcomes	Risk of bias	Inconsistency	Indirectness	Imprecision	Publication Bias	Number of intervention/control	WMD (95%CI)	
TG	No serious limitations	Serious limitation^a^	No serious limitations	No serious limitations	serious limitations	569	−13.85 (−27.21, −0.49)	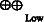
TC	No serious limitations	No serious limitations	No serious limitations	No serious limitations	serious limitations	525	−5.42 (−10.10, −0.75)	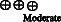
LDL	No serious limitations	No serious limitations	No serious limitations	No serious limitations	No serious limitations	525	−5.29 (−9.63, −0.95)	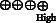
HDL	No serious limitations	No serious limitations	No serious limitations	serious limitations	No serious limitations	569	1.37 (−0.52, 3.27)	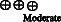
FBS	No serious limitations	No serious limitations	No serious limitations	serious limitations	No serious limitations	430	−2.40 (−5.35, 0.54)	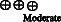
Insulin	No serious limitations	No serious limitations	No serious limitations	serious limitations	No serious limitations	302	0.68 (−0.23, 1.59)	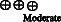
HOMA-IR	No serious limitations	No serious limitations	No serious limitations	serious limitations	No serious limitations	290	0.16 (−0.07, 0.41)	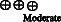
CRP	No serious limitations	Serious limitation^b^	No serious limitations	No serious limitations	No serious limitations	433	−0.01 (−0.05, 0.03)	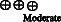
IL-6	No serious limitations	No serious limitations	No serious limitations	serious limitations	No serious limitations	268	−0.68 (−1.55, 0.18)	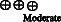
TNF-α	No serious limitations	Very serious limitations^c^	No serious limitations	No serious limitations	serious limitations	241	−2.74 (−4.89, −0.60)	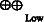
Weight	No serious limitations	No serious limitations	No serious limitations	No serious limitations	serious limitations	397	0.09 (0.06, 0.13)	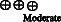
BMI	No serious limitations	Very serious limitations^d^	No serious limitations	serious limitations	No serious limitations	464	−2.69 (−8.74, 3.34)	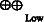
WC	No serious limitations	No serious limitations	No serious limitations	serious limitations	No serious limitations	116	−2.90 (−5.81, 0.00)	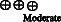
SBP	No serious limitations	No serious limitations	No serious limitations	No serious limitations	No serious limitations	338	−1.37 (−2.73, −0.02)	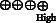
DBP	No serious limitations	No serious limitations	No serious limitations	serious limitations	No serious limitations	338	−0.51 (−1.75, 0.72)	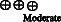

TG, triglyceride; TC, total cholesterol; LDL, low-density lipoprotein; HDL, high- density lipoprotein; FBG, fasting blood glucose; HOMA-IR, homeostasis model assessment for insulin resistance; CRP, C-reactive protein; IL-6, interleukin 6; TNF-α, tumor necrosis factor alpha; BMI, body mass index; WC, waist circumference; SBP, systolic blood pressure; DBP, diastolic blood pressure. ^a^The test for heterogeneity is significant, and the I^2^ is moderate, 69.5%. ^b^The test for heterogeneity is significant, and the I^2^ is moderate, 66.9%. ^c^The test for heterogeneity is significant, and the I^2^ is high, 82.1%. ^d^The test for heterogeneity is significant, and the I^2^ is high, 99.8%.

### 2.5. Statistical analysis

We used Stata version 11.0 (Stata Corp, College Station, TX) to conduct this meta-analysis. We considered all tests to be statistically significant when the *p*-values were less than 0.05; all tests performed were two-tailed. We calculated the pooled weighted mean difference (WMD) using a random effects model developed by Der Simonian & Laird, which was based on the existing heterogeneity. This was due to the different intervention doses, participant health, sample sizes, ethnicity, and length of intervention (38). We calculated the SD of the mean difference using the following formula: SD = square root [(SD at baseline)^2^ + (SD at the end of study)^2^ − (2r × SD at baseline ×SD at the end of study)] (39). Mean differences in CVD risk factors between hesperidin supplementation and control groups were calculated from baseline to post-intervention. SD was calculated using the following formula for RTCs that reported only Standard Error of Mean (SEM): SD = $\mathrm{SEM}\;x\;\sqrt{n}$ , where “n” is the number of participants in each group (40). We used a correlation coefficient of 0.8 for r (41). Heterogeneity was assessed with the I square (I^2^) statistic (*p* < 0.05 and I^2^ > 40%) after visual inspection of forest plots or Cochrane’s Q test (42). A significance level of I^2^ > 40% was considered a clinically important heterogeneity (42). Subgroup analyses were conducted based on baseline LDL (mg/dl) concentration (>130, <130), hesperidin dosage (≤500 mg/d and > 500 mg/d), duration of the intervention (<6 weeks and ≥ 6 weeks), sex (male, both sexes), health status (CVDs, non-CVDs), and baseline BMI [overweight (25–29.9 kg/m^2^) and obese (≥30 kg/m^2^)], age (≥50, <50).

The eager test and the funnel plot test were implemented to assess publication bias in studies evaluating the effects of hesperidin supplementation on CVD risk factors (43). We conducted a sensitivity analysis to determine how many inferences were dependent on a specific sample in order to analyze each study’s effect on the pooled effect size, using the leave-one-out method (removing one trail at a time and recalculating the impact size) (44). Meta-regression was performed for assessing the potential effect of hesperidin (g/d) dosage and duration on CVD risk factors. We also used a non-linear model to synthesize the correlated dose–response data from different studies to assess hesperidin supplementation’s effect on the risk factors of CVDs (45, 46).

### 2.6. Certainty assessment

GRADE (Grading of Recommendations Assessment, Development, and Evaluation) was used to assess and summarize the overall certainty of evidence across the studies (47).

## 3. Meta-analysis

### 3.1. The flow of study selection

**Figure 1:**
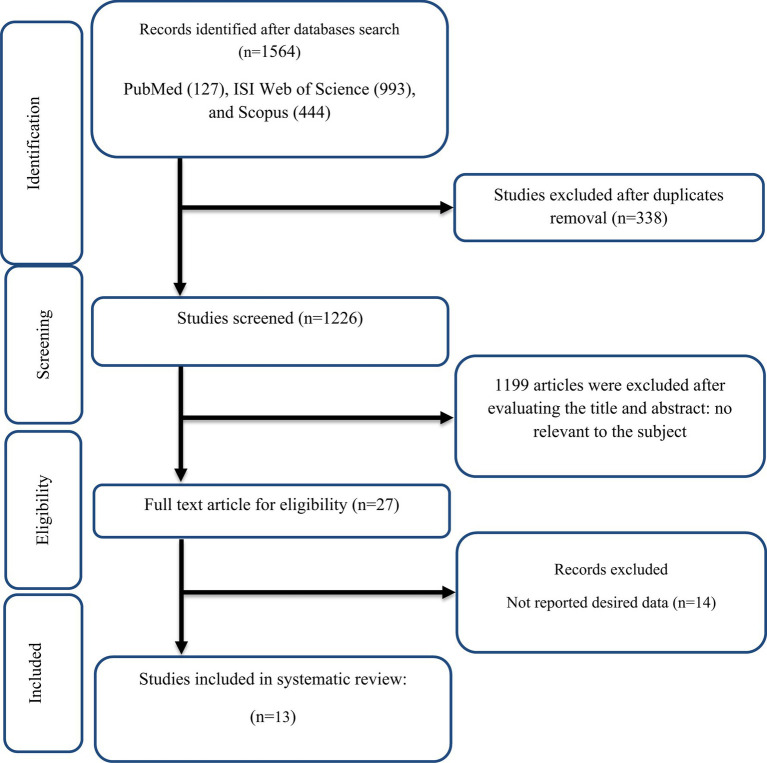
Flow chart of study selection for inclusion trials in the systematic review.

The selection procedure and the references located in the database are described in the flow chart shown in Figure 1. The first round of the literature search of electronic databases yielded a total of 1,564 studies. After removing duplicate studies (*n* = 338) and irrelevant research indicated by titles and abstracts (*n* = 1,199), 27 possibly relevant articles were taken into account for a full-text review. After the screening, 14 studies were excluded due to not reporting the desired data. All of the studies were in the English language. Finally, 13 papers in total were included (23, 28, 29, 48–57) in the meta-analysis. We included ten effect sizes for TG (28, 29, 48–51, 54–57), nine for TC (28, 48–51, 54–57), nine for LDL (28, 48–51, 54–57), ten for HDL (28, 29, 48–51, 54–57), nine for FBG (28, 29, 48–50, 54–57), seven for insulin (28, 29, 48, 50, 54, 55, 57), six for HOMA-IR (28, 29, 48, 50, 52, 57), nine for CRP (23, 28, 48, 50, 51, 53–55, 57), five for IL-6 (23, 50, 51, 53, 54), five for TNF-α (23, 28, 48, 53, 57), seven for weight (28, 49–53, 57), nine for BMI (28, 49–53, 55, 57), three for WC (28, 55, 57), seven for SBP (29, 50, 53–57), seven for DBP (29, 50, 53–57).

### 3.2. Study characteristics

**Figure 2:**
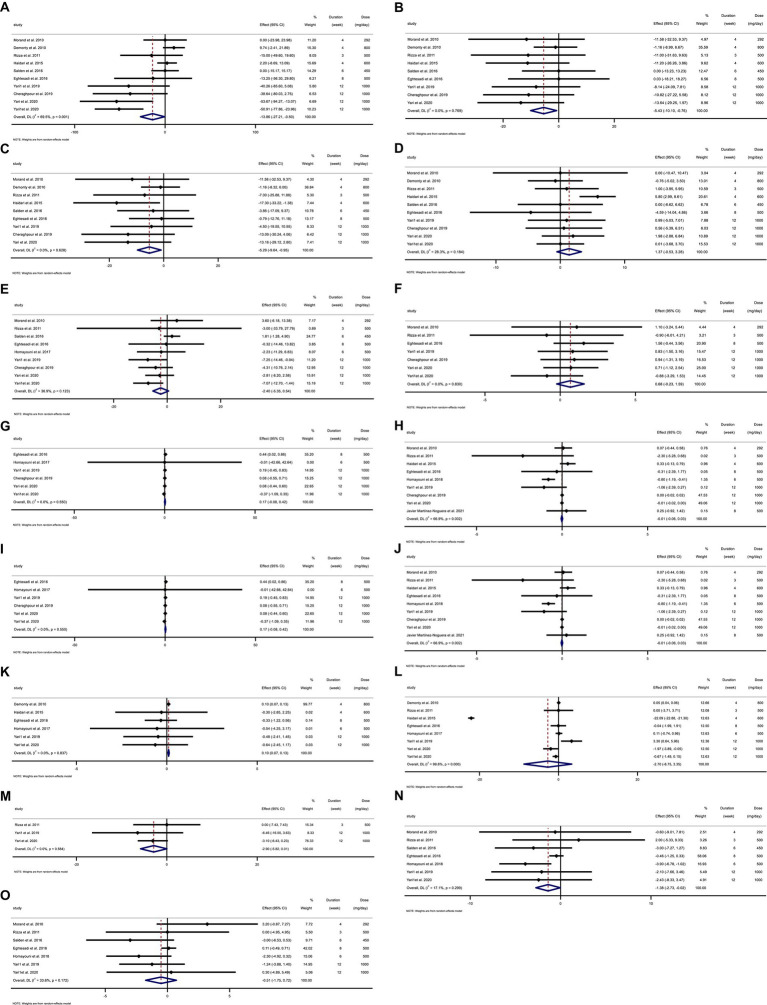
Forest plot detailing weighted mean difference and 95% confidence intervals (CIs) for the effect of hesperidin consumption on **(A)** TG (mg/dL), **(B)** TC (mg/dL), **(C)** LDL (mg/dL), **(D)** HDL (mg/dL), **(E)** FBG (mg/dL), **(F)** fasting insulin (mIU/mL), **(G)** HOMA-IR, **(H)** CRP (mg/L), **(I)** IL-6; (pg/mL), **(J)** TNF-α (pg/mL), **(K)** Weight (kg), **(L)** BMI (kg/m^2^), **(M)** WC (cm), **(N)** SBP (mmHg), and **(O)** DBP (mmHg). Horizontal lines represent 95% of CIs. Diamonds represent pooled estimates from random-effects analysis. WMD, weighted mean difference; CI, confidence interval; TG, triglyceride; TC, total cholesterol; LDL, low-density lipoprotein; HDL, high-density lipoprotein; FBG, fasting blood glucose; HOMA-IR, homeostasis model assessment for insulin resistance; CRP, C-reactive protein; IL-6, interleukin 6; TNF-α, tumor necrosis factor alpha; BMI, body mass index; WC, waist circumference; SBP, systolic blood pressure; DBP, diastolic blood pressure.

Overall, 13 RCTs with 705 individuals were included. These studies were conducted in Iran (28, 29, 48, 50–53, 57), France (54), Italy (55), the Netherlands (49, 56), and Spain (23) and were published between 2010 and 2021. Table 2 lists the characteristics of the study design. Participants’ numbers in these studies ranged from 24 to 124. In the intervention group, the mean age and baseline BMI ranged from 35 (23) to 73 (50) years and 23.1 (23) to 31.7 kg/m^2^ (48), respectively. The WMD and 95% CI of TG (Figure 2A), TC (Figure 2B), LDL (Figure 2C), HDL (Figure 2D), FBG (Figure 2E), fasting insulin (Figure 2F), HOMA-IR (Figure 2G), CRP (Figure 2H), IL-6 (Figure 2I), TNF-α (Figure 2J), weight (Figure 2K), BMI (Figure 2L), WC (Figure 2M), SBP (Figure 2N), and DBP (Figure 2O) were presented in the forest plots. Generally, 12 parallel studies (23, 28, 29, 48–53, 56, 57) and 1 cross-over (54, 55) were conducted. The duration of supplementation ranged from 3 to 12 weeks. Two studies included only male participants (23, 54), and eleven included both sexes (28, 29, 48–53, 55–57). The daily dosage of hesperidin supplementation ranged from 292 mg to 1,000 mg. Studies included participants with hypercholesterolemia (49), type 2 diabetes mellitus (50, 52, 53), metabolic syndrome (29, 55, 57), non-alcoholic fatty liver (28, 48), myocardial infarction (51), those who were overweight but otherwise healthy (54, 56), and amateur cyclists (23).

**Table 2:** Characteristics of included studies in the meta-analysis.

Studies	Country	Study design	Participant	Sample size and sex	Sample size		Trial duration (Week)	Mean age		Mean BMI		Intervention		Adverse events
					IG	CG		IG	CG	IG	CG	Hesperidin dose (mg/d)	Control group	
Morand et al. 2010 (54)	France	Crossover (R, PC, DB)	Healthy overweight individuals	24: 24 M	24	24	4	56 ± 4.89	56 ± 4.89	27.4 ± 1.46	27.4 ± 1.46	292 + 500 mL of the control drink	Placebo +500 mL of the control drink	NR
Demonty et al. 2010 (49)	Netherlands	Parallel (R, PC, DB)	Hypercholesterolemic	124: 59F/65M	59	65	4	61.0 ± 8.6	60.1 ± 8.2	25.1 ± 2.1	25.1 ± 2.3	800	Placebo	Dermatitis in the placebo group (*n* = 1), unspecified gastroduodenitis,
Rizza et al. 2011 (55)	Italy	Crossover (R, PC, DB)	Metabolic Syndrome	24: 9F,15M	12	12	3	53 ± 4.89	50 ± 14.69	33.9 ± 7.62	35.4 ± 6.92	500	Placebo	No adverse effect
Haidari et al. 2015 (51)	Iran	Parallel (R, PC, DB)	Myocardial Infarction	75: 22F,53M	38	37	4	55.49 ± 5.98	55.49 ± 5.98	25.97 ± 2.87	26.82 ± 2.61	600	Placebo	No adverse effect
Salden et al. 2016 (56)	Netherland	Parallel (R, PC, DB)	Healthy overweight individuals	68: 39F,29M	34	34	6	54 ± 15	53 ± 14	28.2 ± 2.2	29.7 ± 2.8	450	Placebo	Skin rash in one participant
Eghtesadi et al. 2016 (50)	Iran	Parallel (R, PC, DB)	Type 2 diabetes	45: 23F, 22 M	23	22	8	73.6 ± 11	73.5 ± 7.49	27 ± 2.58	27.1 ± 3.75	500	Placebo	No adverse effect
Homayouni et al. 2017 (52)	Iran	Parallel (R, PC, DB)	Type 2 diabetes	60: 32F, 28 M	31	29	6	51.26 ± 8.64	54.41 ± 7.84	27.97 ± 2.37	27.49 ± 2.45	500	Placebo	No adverse effect
Homayouni et al. 2018 (53)	Iran	Parallel (R, PC, DB)	Type 2 diabetes	60: 32F, 28 M	31	29	6	51.3 ± 8.6	54.4 ± 7.8	28.0 ± 2.3	27.5 ± 2.4	500	Placebo	No adverse effect
Yari1 et al. 2019 (57)	Iran	Parallel (R, PC, DB)	Metabolic syndrome	49: 24F, 25 M	25	24	12	45.05 ± 11.25	45.33 ± 11.23	29.63 ± 3.80	32.93 ± 5.51	1,000	Placebo	No adverse effect
Cheraghpour et al. 2019 (48)	Iran	Parallel (R, PC, DB)	NAFLD	49: 25F, 24 M	25	24	12	47.32 ± 11.66	47.29 ± 13.76	31.70 ± 5.21	33.00 ± 5.03	1,000	Placebo	NR
Yari et al. 2020 (28)	Iran	Parallel (R, PC, OL)	Non-alcoholic fatty liver	43: 223F, 21 M	22	21	12	45.82 ± 11.69	46.11 ± 11.63	31.07 ± 4.38	33.06 ± 5.14	1,000 + lifestyle modification program	lifestyle modification program	No adverse effect
Yari1 et al. 2020 (29)	Iran	Parallel (R, PC, OL)	metabolic syndrome	44: 23F, 21 M	22	22	12	45.82 ± 11.69	46.41 ± 11.10	29.97 ± 3.40	33.09 ± 5.69	1,000 + lifestyle modification program	Lifestyle modification program	No adverse effect
Javier Martínez-Noguera et al. 2021 (23)	Spain	Parallel (R, PC, DB)	Amateur cyclists	40: 40 M	20	20	8	35.0 ± 9.20	32.6 ± 8.90	23.1 ± 1.53	22.6 ± 1.43	500	Placebo	NR

IG, intervention group; CG, control group; DB, double-blinded; SB, single-blinded; PC, placebo-controlled; CO, controlled; RA, randomized; NR, not reported; F, Female; M, Male; Age, mean age of participants; BMI, mean of body mass index.

### 3.3. Adverse events

Information on adverse effects was mentioned in studies by Demonty et al. (49) (dermatitis in the placebo group and unspecified gastroduodenitis) and Salden et al. (56) (skin rash).

### 3.4. Qualitative data assessment

All 13 studies were rated as being of high quality (Good) using the Cochrane risk of bias assessment method (23, 28, 29, 48–57). There was a total of 10 studies that described the randomization procedure and had a low risk of bias for random sequence generation (23, 28, 48, 51–57). However, others were assigned as having an unclear risk of bias for random sequence generation (29, 49, 50). Due to a lack of clear statements about their methods, two studies were classified as having an unclear risk of bias for allocation concealment (51, 54). Two clinical trials had a high risk of bias for blinding participants and personnel (28, 29). Just one study had a low risk of bias for blinding of outcome assessment (56). In more than 90% of trials, the risk of bias for incomplete outcome data was low. Except for one study (49), every other study was deemed to have a low risk of bias for selective outcome reporting (Table 3).

**Table 3:** Quality assessment (A summary of the risk of bias according to the Cochrane criteria).

Studies	Random sequence generation	Allocation concealment	Selective reporting	Other sources of bias	Blinding (participants and personnel)	Blinding (outcome assessment)	Incomplete outcome data	General risk of bias	Quality
Morand et al. 2010 (54)	L	U	L	U	L	U	L	L	Good
Demonty et al. 2010 (49)	U	L	U	U	L	U	L	L	Good
Rizza et al. 2011 (55)	L	L	L	U	L	U	L	L	Good
Haidari et al. 2015 (51)	L	U	L	L	L	U	U	L	Good
Salden et al. 2016 (56)	L	L	L	U	L	L	L	L	Good
Eghtesadi et al. 2016 (50)	U	L	L	L	L	U	L	L	Good
Homayouni et al. 2017 (52)	L	L	L	L	L	U	L	L	Good
Homayouni et al. 2018 (53)	L	L	L	L	L	U	L	L	Good
Yari1 et al. 2019 (57)	L	L	L	L	L	U	L	L	Good
Cheraghpour et al. 2019 (48)	L	L	L	L	L	U	L	L	Good
Yari et al. 2020 (29)	L	L	L	L	H	U	L	L	Good
Yari1 et al. 2020 (28)	U	L	L	L	H	U	L	L	Good
Javier Martínez-Noguera et al. 2021 (23)	L	L	L	L	L	U	L	L	Good

H, high risk of bias; L, low risk of bias; U, unclear risk of bias. The Cochrane Collaboration tool was used to assess the quality of studies. Bad, <2 low scores; Good, >2 low scores; Fair, 2 Low scores.

### 3.5. Effect of hesperidin supplementation on TG and subgroup analysis

Hesperidin supplementation significantly impacted TG (WMD = −13.85 mg/dL, 95%CI: −27.21, −0.49; *p* = 0.042) (Figure 2A), according to a meta-analysis of 10 studies with a total of 569 participants (28, 29, 48–51, 54–57). The between-study heterogeneity was significant (I^2^ = 69.5%, *p* = 0.001). Hesperidin supplementation significantly decreased TG at doses >500 mg/d (WMD = −23.03 mg/dL, 95%CI: −44.97, −1.09; *p* = 0.040) and duration >6 weeks (WMD = −42.28 mg/dL, 95%CI: −58.96, −25.60; *p* < 0.001), according to subgroup analysis (Table 4). The subgroup analysis showed that TG of the individuals with BMI >30 (WMD = −33.52 mg/dL, 95%CI: −56.34, −10.70; *p* = 0.004), age < 50 years (WMD = −47.41 mg/dL, 95%CI: −65.512, −29.32; *p* < 0.001) and baseline TG >150 (WMD = −37.13 mg/dL, 95%CI: −52.16, −22.11; *p* < 0.001), as well as in studies conducted in both sexes (WMD = −16.20 mg/dL, 95%CI: −31.10, −1.31; *p* = 0.033), generally showed a substantial decline after hesperidin supplementation (Table 4).

**Table 4:** Subgroup analyses of hesperidin supplementation on lipid profiles in adults.

	NO	WMD (95%CI)	*p*-value	Heterogeneity		
				P _heterogeneity_	I^2^	P _between sub-groups_
Subgroup analyses of hesperidin supplementation on TG						
Overall effect	10	−13.85 (−27.21, −0.49)	**0.042**	0.001	69.5%	
Baseline TG (mg/dl)						
<150	4	3.96 (−2.88, 10.82)	0.256	0.721	0.0%	**<0.001**
>150	6	−37.13 (−52.16, −22.11)	**<0.001**	0.491	0.0%	
Trial duration (week)						
≤6	5	3.25 (−3.46, 9.97)	0.343	0.654	0.0%	**<0.001**
>6	5	−42.28 (−58.96, −25.60)	**<0.001**	0.648	0.0%	
Intervention dose (mg/day)						
>500	6	−23.03 (−44.97, −1.09)	**0.040**	<0.001	82.6%	0.107
≤500	4	−2.63 (−14.21, 8.93)	0.655	0.829	0.0%	
Health status						
CVD	5	−13.79 (−34.15, 6.55)	0.184	<0.001	80.0%	0.925
non-CVD	5	−15.14 (−34.53, 4.25)	0.126	0.074	53.0%	
Sex						
Both sexes	9	−16.20 (−31.10, −1.31)	**0.033**	<0.001	72.9%	0.261
Male only	1	0.00 (−23.98, 23.98)	1	-	-	
Baseline BMI (kg/m^2^)						
Overweight (25–29.9)	7	−7.51 (−21.18, 6.15)	0.281	0.003	69.5%	0.055
Obese (>30)	3	−33.52 (−56.34, −10.70)	**0.004**	0.349	5.0%	
Age(year)						
≥50	6	2.86 (−3.78, 9.50)	0.399	0.700	0.0%	**<0.001**
<50	4	−47.41 (−65.512, −29.32)	**<0.001**	0.935	0.0%	
Subgroup analyses of hesperidin supplementation on TC						
Overall effect	9	−5.42 (−10.10, −0.75)	**0.023**	0.769	0.0%	
Baseline TC (mg/dl)						
200≤	4	−3.29 (−9.20, 2.60)	0.274	0.535	0.0%	0.247
200>	5	−9.00 (−16.64, −1.35)	**0.021**	0.848	0.0%	
Trial duration (week)						
≤6	5	−3.88 (−9.55, 1.79)	0.180	0.613	0.0%	0.347
>6	4	−8.68 (−16.91, −0.45)	**0.039**	0.721	0.0%	
Intervention dose (mg/day)						
>500	5	−6.05 (−11.60, −0.50)	**0.033**	0.517	0.0%	0.683
≤500	4	−3.90 (−12.56, 4.75)	0.376	0.686	0.0%	
Health status						
CVD	4	−4.67 (−10.75, 1.41)	0.132	0.565	0.0%	0.704
Non-CVD	5	−6.51 (−13.80, 0.77)	0.080	0.607	0.0%	
Sex						
Both sexes	8	−5.10 (−9.89, −0.31)	**0.037**	0.715	0.0%	0.555
Male only	1	−11.58 (−32.53, 9.37)	0.279	-	-	
Baseline BMI (kg/m^2^)						
Overweight (25–29.9)	6	−3.55 (−8.84, 1.74)	0.189	0.753	0.0%	0.141
Obese (>30)	3	−11.99 (−21.91, −2.08)	**0.018**	0.965	0.0%	
Age						
≥50	6	−3.53 (−8.95, 1.88)	0.201	0.725	0.0%	0.177
<50	3	−10.90 (−20.13, −1.68)	**0.020**	0.890	0.0%	
Subgroup analyses of hesperidin supplementation on LDL						
Overall effect	9	−5.29 (−9.63, −0.95)	**0.017**	0.628	0.0%	
Baseline LDL (mg/dl)						
>130	4	−5.45 (−12.42, 1.52)	0.125	0.288	20.3%	0.824
<130	5	−6.55 (−13.37, 0.25)	0.059	0.703	0.0%	
Trial duration (week)						
≤6	5	−4.64 (−10.04, 0.76)	0.092	0.429	0.0%	0.689
>6	4	−6.49 (−13.80, 0.81)	0.082	0.536	0.0%	
Intervention dose (mg/day)						
>500	5	−7.24 (−13.86, −0.62)	**0.032**	0.272	22.4%	0.543
≤500	4	−4.14 (−11.64, 3.36)	0.279	0.831	0.0%	
Health status						
CVD	4	−4.82 (−11.29, 1.65)	0.145	0.337	11.2%	0.687
non-CVD	5	−6.73 (−13.43, −0.03)	**0.049**	0.647	0.0%	
Sex						
Both sexes	8	−5.01 (−9.45, −0.57)	**0.027**	0.562	0.0%	0.548
Male only	1	−11.58 (−32.53, 9.37)	0.279	-	-	
Baseline BMI (kg/m^2^)						
Overweight (25–29.9)	6	−3.84 (−8.67, 0.98)	0.119	0.540	0.0%	0.178
Obese (>30)	3	−11.43 (−21.36, −1.49)	**0.024**	0.864	0.0%	
Age						
≥50	6	−3.98 (−8.91, 0.93)	0.112	0.526	0.0%	0.269
<50	3	−9.88 (−19.11, −0.65)	**0.036**	0.674	0.0%	
Subgroup analyses of hesperidin supplementation on HDL						
Overall effect	10	1.37 (−0.52, 3.27)	0.157	0.184	28.3%	
Baseline HDL (mg/dl)						
<40	7	1.76 (−0.62, 4.14)	0.781	0.115	41.4%	0.289
>40	3	−0.48 (−3.87, 2.91)	0.147	0.978	0.0%	
Trial duration (week)						
≤6	5	1.84 (−1.43, 5.13)	0.270	0.074	53.1%	0.487
>6	5	0.41 (−1.92, 2.75)	0.726	0.816	0.0%	
Intervention dose (mg/day)						
>500	6	1.75 (−0.75, 4.26)	0.169	0.077	49.7%	0.387
≤500	4	−0.12 (−3.57, 3.32)	0.939	0.787	0.0%	
Health status						
CVD	5	1.68 (−1.22, 4.60)	0.256	0.043	59.4%	0.551
Non-CVD	5	0.42 (−2.54, 3.39)	0.779	0.827	0.0%	
Sex						
Both sexes	9	1.35 (−0.67, 3.37)	0.190	0.133	35.7%	0.804
Male only	1	0.00 (−10.47, 10.47)	1	-	-	
Baseline BMI (kg/m^2^)						
Overweight (25–29.9)	7	1.06 (−1.75, 3.88)	0.460	0.057	50.9%	0.925
Obese (>30)	3	1.26 (−1.73, 4.25)	0.409	0.929	0.0%	
Age						
≥50	6	1.21 (−2.08, 4.51)	0.47	0.055	53.8%	0.822
<50	4	0.74 (−1.67, 3.16)	0.545	0.938	0.0%	

CI, confidence interval; WMD, weighted mean differences; TG, triglyceride; TC, total cholesterol; LDL, low-density lipoprotein; HDL, high- density lipoprotein; BMI, body mass index; CVD, cardiovascular disease. Subgroup analyses have been done. *p* < 0.05 was considered a significance. Bold values are significant.

### 3.6. Effect of hesperidin supplementation on TC and subgroup analysis

The results of the overall analysis of nine studies with 525 participants (28, 48–51, 54–57) revealed that subjects who received hesperidin supplementation had significantly lower TC levels than controls (WMD = −5.42 mg/dL, 95%CI: −10.10, −0.75; *p* = 0.023) (Figure 2B), with no between-study heterogeneity (I^2^ = 0.0%, *p* = 0.769) (Table 4). Hesperidin supplementation caused a greater TC reduction in participants whose baseline TC was less than 200 (WMD = −9.00 mg/dL, 95%CI: −16.64, −1.35; *p* = 0.021), BMI > 30 (WMD = −11.99 mg/dL, 95%CI: −21.91, −2.08; *p* = 0.018), and age < 50 years (WMD = −10.90 mg/dL, 95%CI: −20.13, −1.68; *p* = 0.020) according to the subgroup analysis. Moreover, hesperidin significantly reduced TG at doses of >500 mg/d (WMD = −6.05 mg/dL, 95%CI: −11.60, −0.50; p = 0.033) and duration of more than 6 weeks (WMD = −8.68 mg/dL, 95%CI: −16.91, −0.45; *p* = 0.039) (Table 4), and in studies including both sexes (WMD = −5.10 mg/dL, 95%CI: −9.89, −0.31; *p* = 0.037) (Table 4).

### 3.7. Effect of hesperidin supplementation on LDL and subgroup analysis

The overall analysis of nine studies enrolling 525 participants (28, 48–51, 54–57) showed a significant reduction in LDL levels among those who received hesperidin supplementation compared to the control group (WMD = −5.29 mg/dL, 95%CI: −9.63, −0.95; *p* = 0.017) (Figure 2C), with no between-study heterogeneity (I^2^ = 0.0%, *p* = 0.628). The reduction in LDL after hesperidin supplementation was significant at doses greater than 500, according to the subgroup analysis based on the intervention dose (WMD = −7.24 mg/dL, 95%CI: −13.86, −0.62; *p* = 0.032). Other subgroup analyses also showed that hesperidin significantly reduced the LDL in non-CVD patients (WMD = −6.37 mg/dL, 95%CI: −13.43, −0.03; *p* = 0.049), individuals with BMI > 30 (WMD = −11.43 mg/dL, 95%CI: −21.36, −1.49; *p* = 0.024), age < 50 years (WMD = −9.88 mg/dL; 95%CI: −19.11, −0.65, *p* = 0.036), and in studies conducted in both sexes (WMD = −5.01 mg/dL, 95%CI: −9.45, −0.57; *p* = 0.027) (Table 4).

### 3.8. Effect of hesperidin supplementation on HDL and subgroup analysis

The analysis included 10 trials with a total of 569 participants (28, 29, 48–51, 54–57). The meta-analysis showed an increasing but not statistically significant effect of hesperidin supplementation on HDL (WMD = 1.37 mg/dL, 95% CI: −0.52, 3.27; *p* = 0.157) (Figure 2D), and the heterogeneity was not significant (I^2^ = 28.3%, *p* = 0.184). Our subgroup analyses showed no significant between-study heterogeneity in all subgroups except in studies conducted in patients with CVD (I^2^ = 59.4%, *p* = 0.04) (Table 4).

### 3.9. Effect of hesperidin supplementation on FBG and subgroup analysis

In total, 430 participants from nine studies were included in the analysis (28, 29, 48–50, 54–57). Pooled effect sizes indicated there was not a significant decrease in FBG after supplementation with hesperidin (WMD = −2.40 mg/dL; 95%CI: −5.35, 0.54; *p* = 0.110) (Figure 2E). Between-study heterogeneity was not observed (I^2^ = 36.9%, *p* = 0.123). Furthermore, subgroup analysis showed that hesperidin supplementation affected FBG (WMD = −5.15 mg/dL; 95%CI: −8.17, −2.12; *p* = 0.001) when a high dose of hesperidin (>500 mg/d) was used and the duration of supplementation was longer than 6 weeks (WMD = −4.94 mg/dL; 95%CI: −7.89, −1.98; *p* = 0.001) in individuals with CVD (WMD = −7.05 mg/dL; 95%CI: −11.44, −2.66; *p* = 0.002), age < 50 years (WMD = −5.15 mg/dL; 95%CI: −8.17, −2.12; *p* = 0.001), and in a patient with baseline FBG ≥100 mg/dL (WMD = −4.66 mg/dL; 95%CI: −7.46, −1.86; *p* = 0.001) (Table 5). Subgroup analysis indicated no significant between-study heterogeneity in all subgroups except in overweight participants (I^2^ = 56.4%, *p* = 0.043).

**Table 5:** Subgroup analyses of hesperidin supplementation on insulin and glycemic markers in adults.

	NO	WMD (95%CI)	*p*-value	Heterogeneity		
				P _heterogeneity_	I^2^	P _between sub-groups_
Subgroup analyses of hesperidin supplementation on FBG						
Overall effect	9	−2.40 (−5.35, 0.54)	0.110	0.123	36.9%	
Baseline FBS (mg/dl)						
<100	2	1.97 (−0.97, 4.91)	0.189	0.732	0.0%	**0.001**
≥100	7	−4.66 (−7.46, −1.86)	**0.001**	0.889	0.0%	
Trial duration (week)						
≤6	4	1.53 (−1.25, 4.32)	0.281	0.814	0.0%	**0.002**
>6	5	−4.94 (−7.89, −1.98)	**0.001**	0.737	0.0%	
Intervention dose (mg/day)						
>500	4	−5.15 (−8.17, −2.12)	**0.001**	0.668	0.0%	**0.001**
≤500	5	1.46 (−1.27, 4.20)	0.294	0.908	0.0%	
Health status						
CVD	3	−7.05 (−11.44, −2.66)	**0.002**	0.966	0.0%	**0.005**
Non-CVD	6	0.00 (−2.29, 2.27)	0.994	0.436	0.0%	
Sex						
Both sexes	8	−2.88 (−5.98, 0.20)	0.068	0.115	39.6%	0.215
Male only	1	3.60 (−6.17, 13.37)	0.470	-	-	
Baseline BMI (kg/m^2^)						
Overweight (25–29.9)	6	−2.12 (−6.45, 2.20)	0.337	**0.043**	56.4%	0.669
Obese (>30)	3	−3.42 (−7.51, 0.67)	0.102	0.940	0.0%	
Age						
≥50	5	1.46 (−1.27, 4.20)	0.294	0.908	0.0%	**0.001**
<50	4	−5.15 (−8.17, −2.12)	**0.001**	0.668	0.0%	
Subgroup analyses of hesperidin supplementation on Insulin						
Overall effect	7	0.68 (−0.23, 1.59)	0.145	0.830	0.0%	
Trial duration (week)						
≤6	2	0.26 (−3.04, 3.56)	0.877	0.559	0.0%	0.796
>6	5	0.71 (−0.23, 1.66)	0.141	0.659	0.0%	
Intervention dose (mg/day)						
>500	4	0.46 (−0.61, 1.55)	0.397	0.674	0.0%	0.471
≤500	3	1.21 (−0.49, 2.92)	0.165	0.678	0.0%	
Health status						
CVD	3	−0.08 (−1.67, 1.50)	0.918	0.573	0.0%	0.249
Non-CVD	4	1.05 (−0.06, 2.17)	0.064	0.942	0.0%	
Sex						
Both sexes	6	0.66 (−0.27, 1.59)	0.166	0.732	0.0%	0.846
Male only	1	1.10 (−3.24, 5.44)	0.619	-	-	
Baseline BMI (kg/m^2^)						
Overweight (25–29.9)	4	0.68 (−0.55, 1.91)	0.278	0.492	0.0%	0.999
Obese (>30)	3	0.68 (−0.68, 2.04)	0.330	0.811	0.0%	
Age						
≥50	3	1.21 (−0.49, 2.92)	0.165	0.678	0.0%	0.471
<50	4	0.46 (−0.61, 1.55)	0.397	0.674	0.0%	
Subgroup analyses of hesperidin supplementation on HOMA-IR						
Overall effect	6	0.16 (−0.07, 0.41)	0.180	0.550	0.0%	
Trial duration (week)						
≤6	1	−0.01 (−42.65, 42.63)	1	-	-	0.993
>6	5	0.16 (−0.07, 0.41)	0.180	0.406	0.0%	
Intervention dose (mg/day)						
>500	4	0.02 (−0.28, 0.33)	0.887	0.683	0.0%	0.114
≤500	2	0.44 (0.02, 0.85)	**0.039**	0.984	0.0%	
Health status						
CVD	2	−0.06 (−0.61, 0.48)	0.812	0.253	23.6%	0.311
Non-CVD	4	0.25 (−0.03, 0.54)	0.086	0.686	0.0%	
Baseline BMI (kg/m^2^)						
Overweight (25–29.9)	4	0.18 (−0.19, 0.56)	0.338	0.297	18.7%	0.708
Obese (>30)	2	0.08 (−0.32, 0.48)	0.696	1	0.0%	
Age						
≥50	2	0.44 (0.02, 0.85)	**0.039**	0.984	0.0%	0.114
<50	4	0.02 (−0.28, 0.33)	0.887	0.683	0.0%	

CI, confidence interval; WMD, weighted mean differences; FBG, fasting blood glucose; HOMA-IR, homeostasis model assessment for insulin resistance; BMI, body mass index; CVD, cardiovascular disease. Subgroup analyses have been done. *P* < 0.05 was considered a significance. Bold values are significant.

### 3.10. Effect of hesperidin supplementation on insulin and subgroup analysis

Seven trials with a total of 302 participants (28, 29, 48, 50, 54, 55, 57) were included in the analysis. The meta-analysis showed that hesperidin supplementation did not significantly affect insulin (WMD = 0.68 mIU/mL; 95%CI: −0.23, 1.59; *p* = 0.145) (Figure 2F), and there was no heterogeneity (I^2^ = 0.0%, *p* = 0.830) (Table 5).

### 3.11. Effect of hesperidin supplementation on HOMA-IR and subgroup analysis

Overall, six effect sizes with a total sample size of 290 participants for HOMA-IR were included in the analysis (28, 29, 48, 50, 52, 57). Hesperidin supplementation had not significantly affected HOMA-IR (WMD = 0.16; 95%CI: −0.07, 0.41; *p* = 0.180) (Figure 2G). Between-study heterogeneity was not observed (I^2^ = 0.0%, *p* = 0.550). Subgroup analyses showed that hesperidin supplementation significantly affected HOMA-IR in participants aged ≥50 years (WMD = 0.44; 95%CI: 0.02, 0.85; *p* = 0.039) and in the low dose interventions (≤500 mg/day) (WMD = 0.44; 95%CI: 0.02, 0.85; *p* = 0.039) (Table 5).

### 3.12. Effect of hesperidin supplementation on CRP and subgroup analysis

The overall analysis of nine studies enrolling 433 participants (23, 28, 48, 50, 51, 53–55, 57) indicated no significant changes in CRP among individuals assigned to hesperidin supplementation compared with controls (WMD = −0.01 mg/L; 95%CI: −0.05, 0.03; *p* = 0.560) (Figure 2H), with high between-study heterogeneity (I^2^ = 66.9%, *p* = 0.002) (Table 6). Between-study heterogeneity disappeared in studies with a duration of >6 weeks (I^2^ = 0.0%, *p* = 0.486), those that used >500 mg hesperidin (I^2^ = 42.9%, *p* = 0.154), studies that enrolled obese patients (I^2^ = 34.2%, *p* = 0.219), studies conducted in both sexes (I^2^ = 74.9%%, *p* = 0.001), and when patients were aged <50 years (I^2^ = 10.8%, *p* = 0.339).

**Table 6:** Subgroup analyses of hesperidin supplementation on inflammatory markers in adults.

	NO	WMD (95%CI)	*P*-value	Heterogeneity		
				P _heterogeneity_	I^2^	P _between sub-groups_
Subgroup analyses of hesperidin supplementation on CRP						
Overall effect	9	−0.01 (−0.05, 0.03)	0.560	**0.002**	66.9%	
Trial duration (week)						
≤6	4	−0.25 (−0.97, 0.46)	0.492	0.001	82.6%	0.503
>6	5	0.00 (−0.01, 0.00)	0.243	0.486	0.0%	
Intervention dose (mg/day)						
>500	4	0.00 (−0.02, 0.01)	0.624	0.154	42.9%	0.291
≤500	5	−0.34 (−0.98, 0.28)	0.283	0.041	59.9%	
Health status						
CVD	3	−0.55 (−1.93, 0.81)	0.427	0.042	68.5%	0.438
Non-CVD	6	−0.01 (−0.05, 0.02)	0.528	0.004	71.2%	
Sex						
Both sexes	7	−0.01 (−0.06, 0.03)	0.526	0.001	74.9%	0.633
Male only	2	0.10 (−0.37, 0.57)	0.678	0.778	0.0%	
Baseline BMI (kg/m^2^)						
Normal (18.5–24.9)	1	0.25 (−0.91, 1.42)	0.670	-	-	0.630
Overweight (25–29.9)	5	−0.27 (−0.88, 0.33)	0.382	0.002	76.3%	
Obese (>30)	3	0.00 (−0.02, 0.01)	0.478	0.219	34.2%	
Age						
≥50	5	−0.25 (−0.91, 0.41)	0.458	0.002	76.9%	0.469
<50	4	0.00 (−0.01, 0.00)	0.353	0.339	10.8%	
Subgroup analyses of hesperidin supplementation on IL-6						
Overall effect	5	−0.68 (−1.55, 0.18)	0.121	0.208	32.0%	
Trial duration (week)						
≤6	2	−0.83 (−1.79, 0.11)	0.085	0.128	51.3%	0.240
>6	3	1.26 (−2.11, 4.63)	0.464	0.486	0.0%	
Intervention dose (mg/day)						
>500	1	−2.73 (−4.88, −0.57)	**0.013**	-	-	0.054
≤500	4	−0.53 (−1.08, 0.00)	0.052	0.541	0.0%	
Health status						
CVD	1	−2.73 (−4.88, −0.57)	**0.013**	-	-	
Non-CVD	4	−0.53 (−1.08, 0.00)	0.052	0.541	0.0%	
Sex						
Both sexes	3	−1.14 (−3.06, 0.77)	0.242	0.104	55.8%	0.372
Male only	2	−0.14 (−1.23, 0.94)	0.801	0.674	0.0%	
Baseline BMI (kg/m^2^)						
Normal (18.5–24.9)	1	0.64 (−3.15, 4.43)	0.741	-	-	0.480
Overweight (25–29.9)	4	−0.77 (−1.75, 0.20)	0.122	0.144	44.6%	
Age						
≥50	4	−0.77 (−1.75, 0.20)	0.122	0.144	44.6%	0.480
<50	1	0.64 (−3.15, 4.43)	0.741	-	-	
Subgroup analyses of hesperidin supplementation on TNF-α						
Overall effect	5	−2.74 (−4.89, −0.60)	**0.012**	<0.001	82.1%	
Trial duration (week)						
≤6	1	−2.30 (−4.13, −0.46)	**0.014**	-	-	0.673
>6	4	−3.03 (−5.92, −0.14)	**0.039**	<0.001	84.8%	
Intervention dose (mg/day)						
>500	3	−3.93 (−5.57, −2.28)	**<0.001**	0.412	0.0%	0.038
≤500	2	−0.99 (−3.22, 1.24)	0.384	0.024	80.5%	
Health status						
CVD	1	−3.15 (−5.29, −1.00)	**0.004**	-	-	0.788
Non-CVD	4	−2.69 (−5.24, −0.14)	**0.039**	0.001	83.0%	
Sex						
Both sexes	4	−3.28 (−4.64, −1.93)	**<0.001**	0.327	13.1%	**<0.001**
Male only	1	0.00 (−0.76, 0.76)	1	-	-	
Baseline BMI (kg/m^2^)						
Normal (18.5–24.9)	1	0.00 (−0.76, 0.76)	1	-	-	**<0.001**
Overweight (25–29.9)	2	−2.66 (−4.05, −1.26)	**<0.001**	0.555	0.0%	
Obese (>30)	2	−5.06 (−7.63, −2.48)	**<0.001**	0.470	0.0%	
Age						
≥50	1	−2.30 (−4.13, −0.46)	**0.014**	-	-	0.673
<50	4	−3.03 (−5.92, −0.14)	**0.039**	<0.001	84.8%	

CI, confidence interval; WMD, weighted mean differences; CRP, C-reactive protein; IL-6, interleukin 6; TNF-α, tumor necrosis factor alpha; BMI, body mass index; CVD, cardiovascular disease. Subgroup analyses have been done. *P* < 0.05 was considered a significance. Bold values are significant.

### 3.13. Effect of hesperidin supplementation on IL-6 and subgroup analysis

The meta-analysis of five studies with a total of 268 participants (23, 50, 51, 53, 54) demonstrated that hesperidin supplementation did not significantly affect IL-6 (WMD = −0.68 pg./mL; 95%CI: −1.55, 0.18; *p* = 0.121) (Figure 2I). The between-study heterogeneity was not significant (I^2^ = 32.0%, *p* = 0.208). In subgroup analysis, we observed that hesperidin significantly reduced IL-6 at doses of >500 mg/d (WMD = −2.73 pg./mL; 95%CI: −4.88, −0.57; *p* = 0.013) and in patients with CVD (WMD = −2.73 pg./mL; 95%CI: −4.88, −0.57; *p* = 0.013) (Table 6).

### 3.14. Effect of hesperidin supplementation on TNF-α and subgroup analysis

Overall, five effect sizes with a total of 241 participants (23, 28, 48, 53, 57) demonstrated a considerable decrease in TNF-α among subjects assigned to hesperidin supplementation compared with controls (WMD = −2.74 pg./mL; 95%CI: −4.89, −0.60; *p* = 0.012) (Figure 2J). Between-study heterogeneity was also observed (I^2^ = 82.1%, *p* < 0.001). After subgroup analysis, we found that between-study heterogeneity disappeared in studies with intervention dose >500 (I^2^ = 0.0%, *p* = 0.412), studies conducted in both sexes (I^2^ = 13.1%, *p* = 0.327), and studies that enrolled obese (I^2^ = 0.0%, *p* = 0.470) and overweight (I^2^ = 0.0%, *p* = 0.555) individuals. Additionally, subgroup analyses revealed that hesperidin supplementation significantly reduced TNF-α in every subgroup except for those who received an intervention dose of ≤500 (WMD = −0.99 pg./mL; 95%CI: −3.22, 1.24; *p* = 0.384), those who were of normal weight (18.5 < BMI < 24.9) (WMD = 0.0 pg./mL; 95%CI: −0.76, 0.76; *p* = 1), and in studies conducted in men only (WMD = 0.0 pg./mL; 95%CI: −0.76, 0.76; *p* = 1) (Table 6).

### 3.15. Effect of hesperidin supplementation on weight and subgroup analysis

To assess how hesperidin supplementation affected weight, seven studies with a combined sample size of 457 participants were taken into consideration (28, 49–53, 57). We discovered a significant effect of hesperidin supplementation on weight (WMD = 0.09 kg; 95%CI: 0.06, 0.13, *p* < 0.001) by combining the data from these investigations (Figure 2K). No between-study heterogeneity existed (I^2^ = 0.0%; *p* = 0.902). Subgroup analyses revealed that hesperidin supplementation had a significant effect on weight in participants aged ≥50 (WMD = 0.09 kg; 95%CI: 0.06, 0.13, p < 0.001), in the high dose interventions (>500) (WMD = 0.09 kg; 95%CI: 0.06, 0.13, p < 0.001), in trial duration ≤6 weeks (WMD = 0.10 kg; 95%CI: 0.06, 0.13, *p* < 0.001), and in patients with CVD (WMD = 0.09 kg; 95%CI: 0.06, 0.13, *p* < 0.001) (Table 7).

**Table 7:** Subgroup analyses of hesperidin supplementation on anthropometric measurements in adults.

	NO	WMD (95%CI)	*p*-value	Heterogeneity		
				P _heterogeneity_	I^2^	P _between sub-groups_
Subgroup analyses of hesperidin supplementation on weight						
Overall effect	6	0.09 (0.06, 0.13)	**<0.001**	0.837	0.0%	
Trial duration (week)						
≤6	3	0.10 (0.06, 0.13)	**<0.001**	0.901	0.0%	0.182
>6	3	−0.40 (−1.14, 0.33)	0.284	0.952	0.0%	
Intervention dose (mg/day)						
>500	4	0.09 (0.06, 0.13)	**<0.001**	0.781	0.0%	0.319
≤500	2	−0.34 (−1.20, 0.52)	0.440	0.914	0.0%	
Health status						
CVD	4	0.09 (0.06, 0.13)	**<0.001**	0.781	0.0%	0.319
Non-CVD	2	−0.34 (−1.20, 0.52)	0.440	0.914	0.0%	
Age						
≥50	4	0.09 (0.06, 0.13)	**<0.001**	0.777	0.0%	0.324
<50	2	−0.56 (−1.88, 0.75)	0.402	0.906	0.0%	
Subgroup analyses of hesperidin supplementation on BMI						
Overall effect	8	−2.69 (−8.74, 3.34)	0.38	**<0.001**	99.8%	
Baseline BMI (kg/m^2^)						
Overweight (25–29.9)	6	−3.25 (−10.44, 3.94)	0.376	**<0.001**	99.8%	0.653
Obese (>30)	2	−1.55 (−3.26, 0.15)	0.074	0.356	0.0%	
Trial duration (week)						
≤6	4	−5.51 (−16.64, 5.61)	0.331	**<0.001**	99.8%	0.345
>6	4	−0.10 (−1.71, 1.50)	0.898	**0.015**	71.3%	
Intervention dose (mg/day)						
>500	5	−4.30 (−13.61, 5.01)	0.365	**<0.001**	99.9%	0.358
≤500	3	0.08 (−0.67, 0.84)	0.832	0.990	0.0%	
Health status						
CVD	5	−3.92 (−13.52, 5.67)	0.423	**<0.001**	99.9%	0.481
Non-CVD	3	−0.44 (−1.64, 0.75)	0.470	0.150	47.3%	
Age						
≥50	5	−4.42 (−13.97, 5.12)	0.364	**<0.001**	99.9%	0.380
<50	3	−0.02 (−2.29, 2.23)	0.981	**0.006**	80.4%	
Subgroup analyses of hesperidin supplementation on WC						
Overall effect	3	−2.90 (−5.81, 0.00)	**0.051**	0.584	0.0%	

CI, confidence interval; WMD, weighted mean differences; BMI, body mass index; WC, waist circumference; BMI, body mass index; CVD, cardiovascular disease. Subgroup analyses have been done. *p* < 0.05 was considered a significance. Bold values are significant.

### 3.16. Effect of hesperidin supplementation on BMI and subgroup analysis

Across the nine effect sizes involving a total of 524 participants, there was very little difference in BMI between the intervention group and control group (28, 29, 49–53, 55, 57) (WMD = −2.39 kg/m^2^, 95%CI: −7.45, 2.66; *p* = 0.35) (Figure 2L). Between-study heterogeneity was observed (I^2^ = 99.7%, *p* < 0.001) (Table 7).

### 3.17. Effect of hesperidin supplementation on WC and subgroup analysis

The analysis of three trials with a total of 116 participants (28, 55, 57), which provided data on WC changes, revealed that participants in the hesperidin supplementation group had a WC reduction of 2.90 cm more than those in the control group (Figure 2M), but this difference was not statistically significant (95% CI: −5.81, 0.00; *p* = 0.051). The test for between-study heterogeneity was not significant (I^2^ = 0.0%, *p* = 0.584) (Table 7).

### 3.18. Effect of hesperidin supplementation on SBP and subgroup analysis

Our meta-analysis included seven clinical trials (29, 50, 53–57) with a total of 338 participants. Combining these effect sizes, we found a significant effect of hesperidin supplementation on SBP (WMD = −1.37 mmHg; 95% CI: −2.73, −0.02; *p* = 0.046) (Figure 2N), with no significant between-study heterogeneity. Subgroup analysis revealed that duration of intervention, baseline BMI, and sex accounted for the between-study heterogeneity. We observed a significant effect of hesperidin supplementation on SBP in studies that included both men and women (WMD = −1.60 mmHg; 95%CI: −3.23, −0.03; *p* = 0.055), overweight patients (25 < BMI < 29.9) (WMD = −1.63 mmHg; 95%CI: −3.15, −0.10; *p* = 0.036), and those with a duration of intervention ≤6 weeks (WMD = −2.91 mmHg; 95%CI: −5.10, −0.71; *p* = 0.009) (Table 8).

**Table 8:** Subgroup analyses of hesperidin supplementation on blood pressure in adults.

	NO	WMD (95%CI)	*p*-value	Heterogeneity		
				P _heterogeneity_	I^2^	P _between sub-groups_
Subgroup analyses of hesperidin supplementation on SBP						
Overall effect	7	−1.37 (−2.73, −0.02)	**0.046**	0.299	17.1%	
Trial duration (week)						
≤6	4	−2.91 (−5.10, −0.71)	**0.009**	0.481	0.0%	0.044
>6	3	−0.52 (−1.30, 0.24)	0.183	0.693	0.0%	
Intervention dose (mg/day)						
≤500	5	−1.543 (−3.49, 0.40)	0.121	0.152	40.4%	0.756
>500	2	−2.25 (−6.30, 1.79)	0.275	0.936	0.0%	
Health status						
CVD	3	−1.26 (−4.80, 2.28)	0.485	0.607	0.0%	0.780
Non-CVD	4	−1.85 (−4.04, 0.32)	0.096	0.104	51.4%	
Sex						
Both sexes	6	−1.60 (−3.23, 0.03)	**0.055**	0.203	30.9%	0.819
Male only	1	−0.60 (−9.00, 7.80)	0.889	-	-	
Baseline BMI (kg/m^2^)						
Overweight (25–29.9)	6	−1.63 (−3.15, −0.10)	**0.036**	0.246	25.1%	0.342
Obese (>30)	1	2.00 (−5.33, 9.33)	0.593	-	-	
Age						
≥50	5	−1.54 (−3.49, 0.40)	0.121	0.152	40.4%	0.756
<50	2	−2.25 (−6.30, 1.79)	0.275	0.936	0.0%	
Subgroup analyses of hesperidin supplementation on DBP						
Overall effect	7	−0.51 (−1.75, 0.72)	0.415	0.172	33.6%	
Trial duration (week)						
≤6	4	−0.81 (−3.49, 1.87)	0.553	0.094	53.1%	0.539
>6	3	0.04 (−0.52, 0.62)	0.871	0.616	0.0%	
Intervention dose (mg/day)						
≤500	5	−0.50 (−2.23,1.22)	0.566	0.084	51.4%	0.779
>500	2	−0.92 (−3.27, 1.42)	0.441	0.604	0.0%	
Health status						
CVD	3	−0.75 (−2.87, 1.36)	0.486	0.828	0.0%	0.908
Non-CVD	4	−0.58 (−2.61, 1.45)	0.575	0.042	63.5%	
Sex						
Both sexes	6	−0.66 (−1.76, 0.43)	0.233	0.261	23.1%	0.072
Male only	1	3.20 (−0.86, 7.26)	0.123	-	-	
Baseline BMI (kg/m^2^)						
Overweight (25–29.9)	6	−0.58 (−1.99, 0.81)	0.413	0.108	44.6%	0.823
Obese (>30)	1	0.00 (−4.95, 4.95)	1	-	-	
Age						
≥50	5	−0.50 (−2.23, 1.22)	0.566	0.084	51.4%	0.779
<50	2	−0.92 (−3.27, 1.42)	0.441	0.604	0.0%	

CI, confidence interval; WMD, weighted mean differences; BMI, body mass index; SBP, systolic blood pressure; DBP, diastolic blood pressure; BMI, body mass index; CVD, cardiovascular disease. Subgroup analyses have been done. *p* < 0.05 was considered a significance. Bold values are significant.

### 3.19. Effect of hesperidin supplementation on DBP and subgroup analysis

We obtained data on changes in DBP from seven trials, which included a total of 338 participants (29, 50, 53–57). The random-effects meta-analysis indicated that compared with control, hesperidin supplementation did not significantly reduce DBP (WMD = −0.51 mmHg; 95%CI: −1.75, 0.72; *p* = 0.415) (Figure 2O) and the between-study heterogeneity was not significant (I^2^ = 33.6%, *p* = 0.172) (Table 8).

### 3.20. Non-linear dose–response analysis

**Figure 3:**
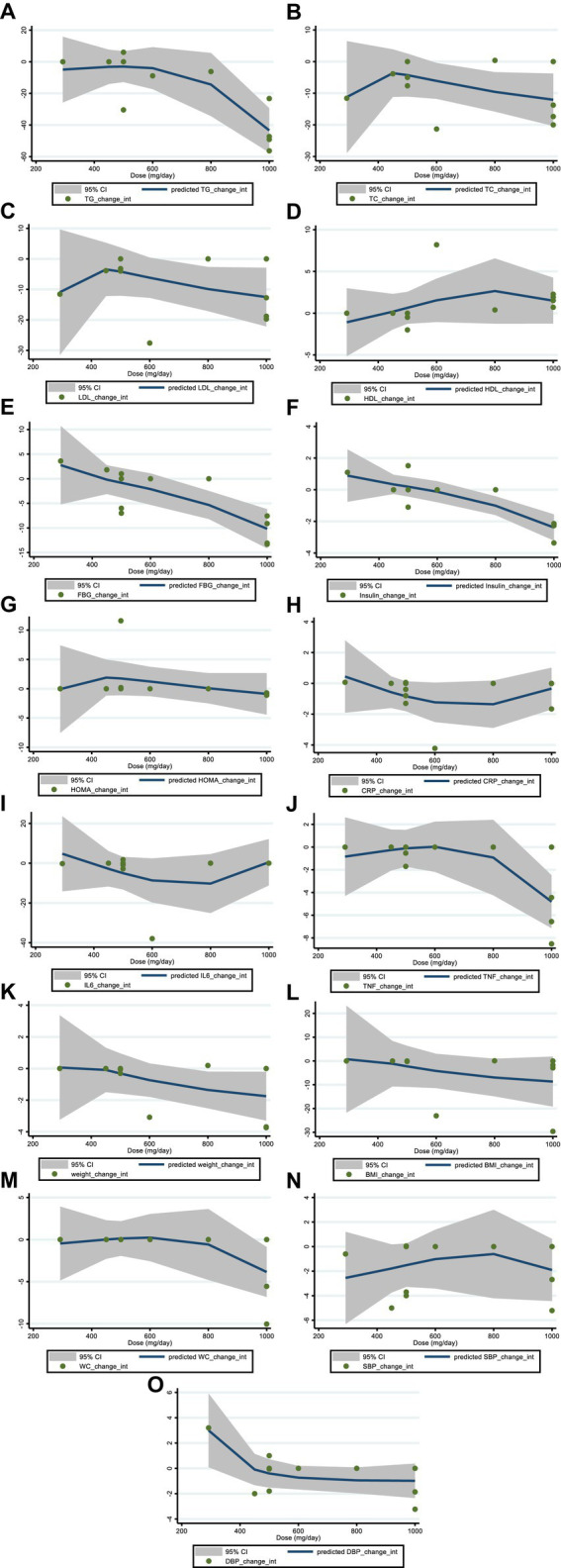
Non-linear dose–response analysis on effects of hesperidin dosage (mg/d) on **(A)** TG (mg/dL), **(B)** TC (mg/dL), **(C)** LDL (mg/dL), **(D)** HDL (mg/dL), **(E)** FBG (mg/dL), **(F)** fasting insulin (mIU/mL), **(G)** HOMA-IR, **(H)** CRP (mg/L), **(I)** IL-6; (pg/mL), **(J)** TNF-α (pg/mL), **(K)** Weight (kg), **(L)** BMI (kg/m^2^), **(M)** WC (cm), **(N)** SBP (mmHg), and **(O)** DBP (mmHg). TG, triglyceride; TC, total cholesterol; LDL, low-density lipoprotein; HDL, high-density lipoprotein; FBG, fasting blood glucose; HOMA-IR, homeostasis model assessment for insulin resistance; CRP, C-reactive protein; IL-6, interleukin 6; TNF-α, tumor necrosis factor alpha; BMI, body mass index; WC, waist circumference; SBP, systolic blood pressure; DBP, diastolic blood pressure; CI, confidence interval.

**Figure 4:**
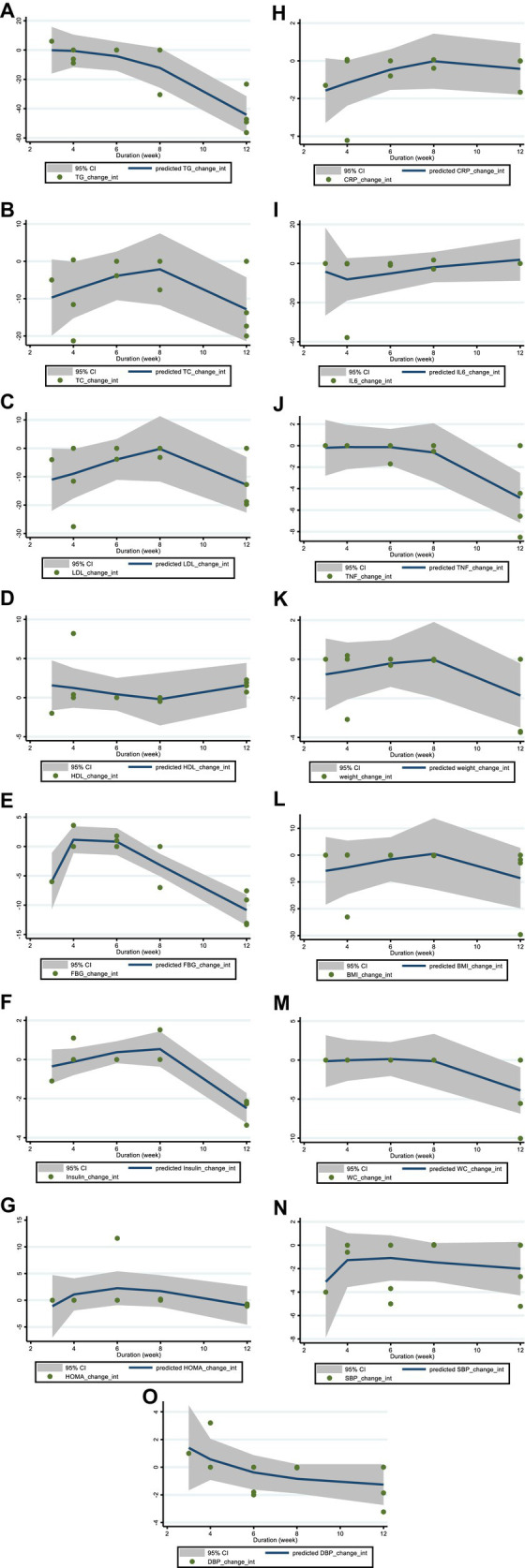
Non-linear dose–response analysis on effects of duration of the intervention (week) on **(A)** TG (mg/dL), **(B)** TC (mg/dL), **(C)** LDL (mg/dL), **(D)** HDL (mg/dL), **(E)** FBG (mg/dL), **(F)** fasting insulin (mIU/mL), **(G)** HOMA-IR, **(H)** CRP (mg/L), **(I)** IL-6; (pg/mL), **(J)** TNF-α (pg/mL), **(K)** Weight (kg), **(L)** BMI (kg/m^2^), **(M)** WC (cm), **(N)** SBP (mmHg), and **(O)** DBP (mmHg). TG, triglyceride; TC, total cholesterol; LDL, low-density lipoprotein; HDL, high-density lipoprotein; FBG, fasting blood glucose; HOMA-IR, homeostasis model assessment for insulin resistance; CRP, C-reactive protein; IL-6, interleukin 6; TNF-α, tumor necrosis factor alpha; BMI, body mass index; WC, waist circumference; SBP, systolic blood pressure; DBP, diastolic blood pressure; CI, confidence interval.

We utilized a non-linear dose–response regression to analyze the dose–response relationship between hesperidin supplementation and cardiovascular risk variables. Hesperidin dose and insulin levels were shown to have a non-linear relationship (coefficients = −0.002, P _non-linearity_ = 0.004; Figure 3F), with a dosage of approximately 1,000 mg/d of hesperidin causing the greatest decrease in insulin levels. Moreover, there was a non-linear association between the duration of intervention and insulin, with the highest reduction after 8 weeks (coefficients = 0.01, P _non-linearity_ = 0.001; Figure 4F). In addition, the results have shown a non-linear association between the duration of intervention and FBG with the reduction after 6 weeks (coefficients = −0.145, P _non-linearity_ = 0.001; Figure 3E). However, we did not observe a significant effect of supplementation dosage on TG (P _non-linearity_ = 0.551; Figure 3A), TC (P _non-linearity_ = 0.173; Figure 3B), LDL (P _non-linearity_ = 0.221; Figure 3C), HDL (P _non-linearity_ = 0.341; Figure 3D), HOMA-IR (P _non-linearity_ = 0.313; Figure 3G), TNF-α (P _non-linearity_ = 0.447; Figure 3J), IL-6 (P _non-linearity_ = 0.233; Figure 3I), CRP (P _non-linearity_ = 0.0.231; Figure 3H), BMI (P _non-linearity_ = 0.696; Figure 3L), weight (P _non-linearity_ = 0.502; Figure 3K), WC (P _non-linearity_ = 0.616; Figure 3M), SBP (P _non-linearity_ = 0.501; Figure 3N), and DBP (P _non-linearity_ = 0.248; Figure 3O).

### 3.21. Meta-regression analysis

**Figure 5:**
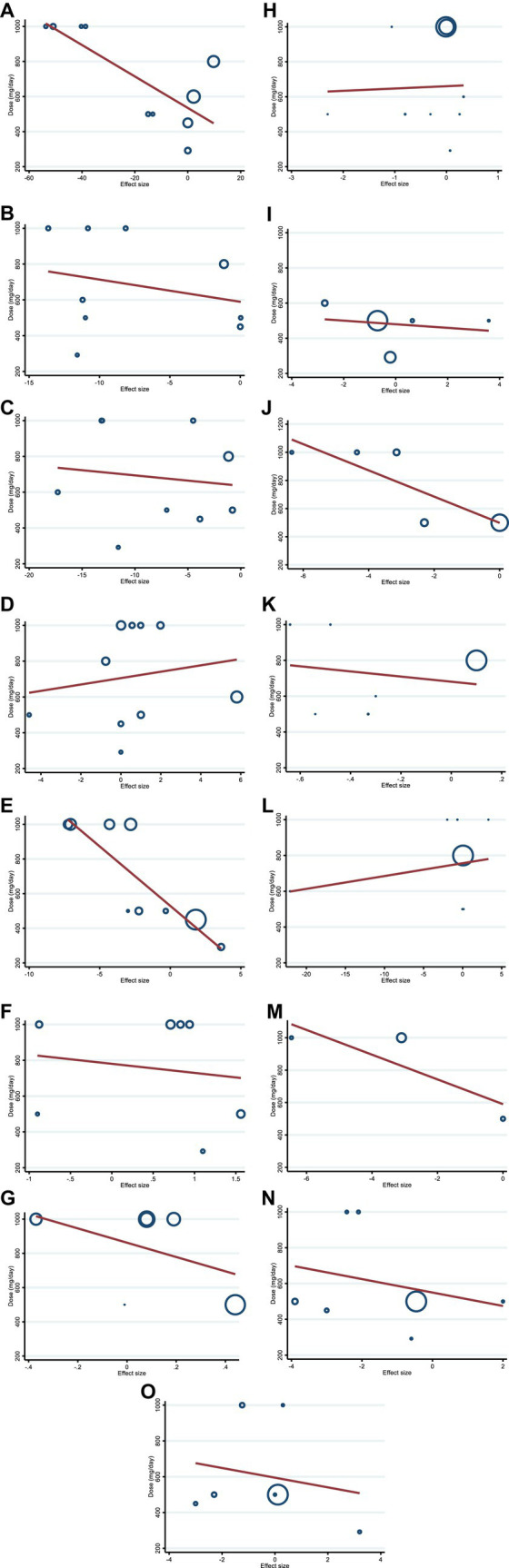
Random-effects meta-regression plots of the association between the dose of hesperidin (mg/day) and weighted mean difference of **(A)** TG (mg/dL), **(B)** TC (mg/dL), **(C)** LDL (mg/dL), **(D)** HDL (mg/dL), **(E)** FBG (mg/dL), **(F)** fasting insulin (mIU/mL), **(G)** HOMA-IR, **(H)** CRP (mg/L), **(I)** IL-6; (pg/mL), **(J)** TNF-α (pg/mL), **(K)** Weight (kg), **(L)** BMI (kg/m^2^), **(M)** WC (cm), **(N)** SBP (mmHg), and **(O)** DBP (mmHg). TG, triglyceride; TC, total cholesterol; LDL, low-density lipoprotein; HDL, high-density lipoprotein; FBG, fasting blood glucose; HOMA-IR, homeostasis model assessment for insulin resistance; CRP, C-reactive protein; IL-6, interleukin 6; TNF-α, tumor necrosis factor alpha; BMI, body mass index; WC, waist circumference; SBP, systolic blood pressure; DBP, diastolic blood pressure.

**Figure 6:**
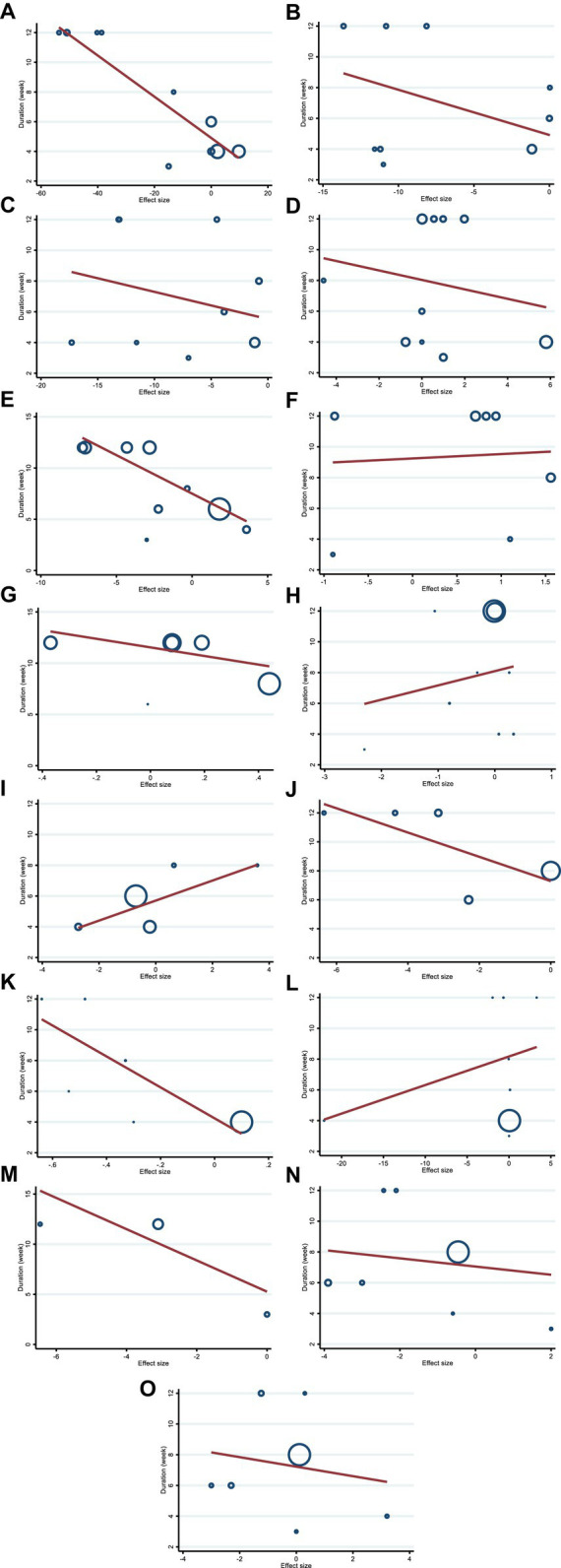
Random-effects meta-regression plots of the association between duration of intervention and weighted mean difference of **(A)** TG (mg/dL), **(B)** TC (mg/dL), **(C)** LDL (mg/dL), **(D)** HDL (mg/dL), **(E)** FBG (mg/dL), **(F)** fasting insulin (mIU/mL), **(G)** HOMA-IR, **(H)** CRP (mg/L), **(I)** IL-6; (pg/mL), **(J)** TNF-α (pg/mL), **(K)** Weight (kg), **(L)** BMI (kg/m^2^), **(M)** WC (cm), **(N)** SBP (mmHg), and **(O)** DBP (mmHg). TG, triglyceride; TC, total cholesterol; LDL, low-density lipoprotein; HDL, high-density lipoprotein; FBG, fasting blood glucose; HOMA-IR, homeostasis model assessment for insulin resistance; CRP, C-reactive protein; IL-6, interleukin 6; TNF-α, tumor necrosis factor alpha; BMI, body mass index; WC, waist circumference; SBP, systolic blood pressure; DBP, diastolic blood pressure.

We conducted a meta-regression analysis to assess the impact of hesperidin doses and intervention duration on cardiovascular risk variables. We discovered a significant linear association between the intervention’s dose (g/day) (coefficients = −68.62, P _linearity_ = 0.004) (Figure 5E) and duration of the intervention (weeks) (coefficients = −0.75, *p* = 0.032) (Figure 6E) and changes in FBG. In addition, there was a significant linear relationship between the dose of intervention of hesperidin and TG changes (coefficients = −8.97, *p* = 0.009) (Figure 5A). There was no significant linear relationship between dose and duration of intervention and changes in other variables.

### 3.22. Sensitivity analysis

Our sensitivity analysis revealed that the effect sizes for the impact of hesperidin on TG, TC, LDL, HDL, insulin, HOMA-IR, CRP, IL-6, BMI, weight, SBP, and DBP remained robust even after removing each study one by one. While based on the results of sensitivity analysis, data on FBG was sensitive to studies by Salden et al. (56) (WMD = −4.03, 95%CI: −6.73, −1.34), and the overall results were changed to significant. The overall effect of hesperidin on WC also changed to a significant value after excluding the study by Rizza et al. (55) (WMD = −3.43, 95%CI: −6.59, −0.26).

### 3.23. GRADE assessment

Table 1 displays the GRADE profile of hesperidin supplementation on cardiovascular risk variables together with the certainty in outcomes. For LDL and SBP, the quality of the evidence was good. The quality of the evidence was moderate for DBP, WC, weight, IL-6, CRP, HOMA-IR, insulin, FBG, HDL, and TC but low for TG, TNF-α, and BMI.

### 3.24. Publication bias

**Figure 7:**
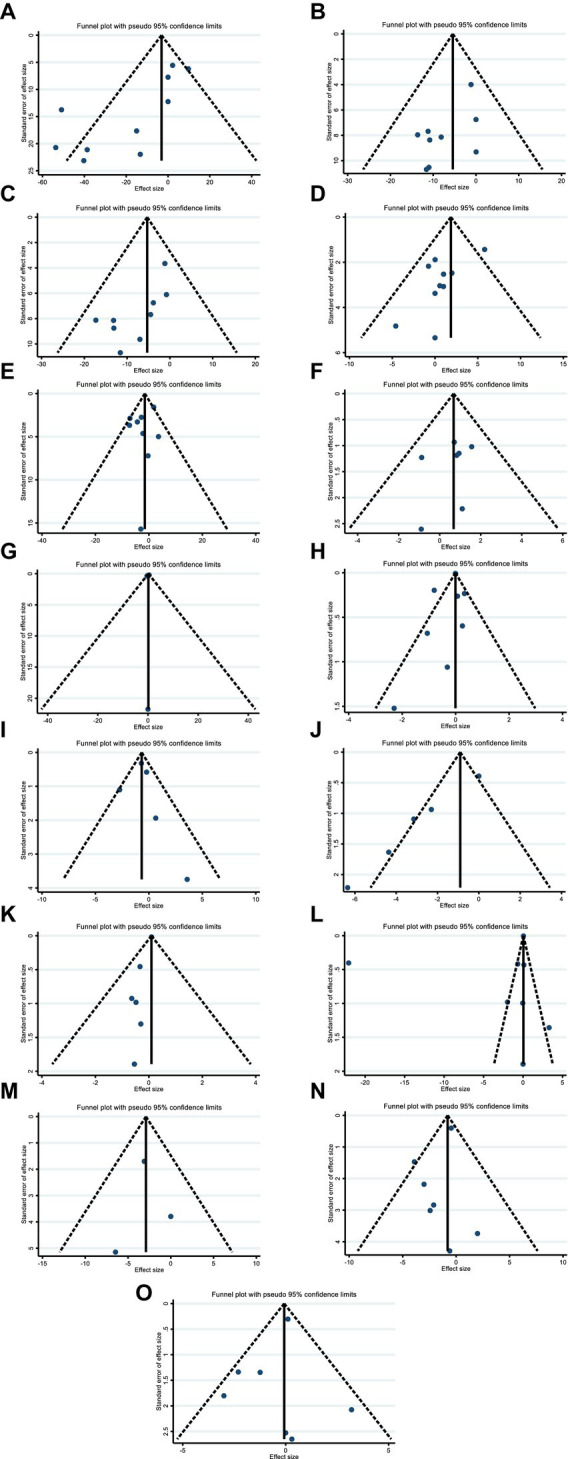
Funnel plots for the effect of hesperidin consumption on **(A)** TG (mg/dL), **(B)** TC (mg/dL), **(C)** LDL (mg/dL), **(D)** HDL (mg/dL), **(E)** FBG (mg/dL), **(F)** fasting insulin (mIU/mL), **(G)** HOMA-IR, **(H)** CRP (mg/L), **(I)** IL-6; (pg/mL), **(J)** TNF-α (pg/mL), **(K)** Weight (kg), **(L)** BMI (kg/m^2^), **(M)** WC (cm), **(N)** SBP (mmHg), and **(O)** DBP (mmHg). TG, triglyceride; TC, total cholesterol; LDL, low-density lipoprotein; HDL, high-density lipoprotein; FBG, fasting blood glucose; HOMA-IR, homeostasis model assessment for insulin resistance; CRP, C-reactive protein; IL-6, interleukin 6; TNF-α, tumor necrosis factor alpha; BMI, body mass index; WC, waist circumference; SBP, systolic blood pressure; DBP, diastolic blood pressure; CI, confidence interval.

Although the visual inspection of funnel plots showed slight asymmetries in publication bias, Egger’s test indicated no significant evidence of bias in the meta-analysis for the effect of hesperidin supplementation on TC (*p* = 0.052) (Figure 7B), LDL (*p* = 0.159) (Figure 7C), FBG (*p* = 0.365) (Figure 7E), insulin (*p* = 0.463) (Figure 7F), HOMA-IR (*p* = 0.492) (Figure 7G), CRP (*p* = 0.307) (Figure 7H), IL-6 (*p* = 0.764) (Figure 7I), BMI (*p* = 0.343) (Figure 7L), WC (*p* = 0.968) (Figure 7M), SBP (*p* = 0.274) (Figure 7N), and DBP (*p* = 0.486) (Figure 7O); however, the Egger’s test showed significant asymmetry for TG (*p* = 0.007) (Figure 7A), HDL (*p* = 0.035) (Figure 7D), weight (*p* = 0.005) (Figure 7K), and TNF-α (*p* = 0.001) (Figure 7J).

## 4. Discussion

We conducted a comprehensive systematic review and meta-analysis to examine the effects of hesperidin supplementation on cardiovascular risk factors in adults. Our analysis investigated the impact of hesperidin on various biomarkers associated with CVD risk, including the lipid profile, inflammatory markers, blood glucose and insulin, blood pressure, and weight. While some previous systematic reviews have examined the effects of hesperidin, none have analyzed all of these biomarkers, and the results have not been conclusive. Our analysis revealed that hesperidin supplementation significantly reduces TG, TC, LDL, TNF-α, and SBP, while also causing weight gain in participants.

Hesperidin belongs to flavanones, a class of polyphenols that are a group of bioactive plant compounds and have been shown to *have positive effects on cardiovascular health* (20, 58). *It is found in high amounts in citrus fruits and juices* (59). Absorption of this compound happens in the gastrointestinal tract, specifically in the colon, since hesperidin is resistant to enzymes produced in the stomach and small intestine. The intestinal microbiota breaks hesperidin and converts it to the aglycone form, which is named hesperetin. This can happen through one-step deglycosylation by α-rhamnosyl-β- glucosidase or through two-step deglycosylation by α-rhamnosidase and β-glucosidase. Eventually, hesperetin is absorbed through the intestinal epithelium and released into the bloodstream in the form of glucuronide and sulfate conjugates (60, 61). Considering the abovementioned mechanism, *the bioavailability and biological effects of hesperidin are influenced by the form in which it is ingested and the composition of the intestinal microbiota* (13).

The current meta-analysis found that hesperidin supplementation significantly reduces serum TG, TC, and LDL levels in adults. A systematic review conducted by Tadros et al. in 2021 analyzed the impact that hesperidin in 100% orange juice (hesperidin is one of the components in orange juice and other components may also be involved in its effect) has on chronic disease biomarkers in humans over 18 years of age and found results that were not conclusive about HDL or LDL (62). In 2019, Pla-Paga et al. conducted another systematic review to assess the impact of hesperidin consumption on cardiovascular risk biomarkers in both animal studies and human randomized clinical trials. The review found that hesperidin had beneficial effects in reducing TC, LDL, and TG in animal models. However, in human studies, no consensus was reached regarding hesperidin’s effect on cardiovascular risk biomarkers. While TC, LDL, HDL, and TG were evaluated in human studies, no significant changes were observed in any of these biomarkers (63). In addition, a systematic review and meta-analysis carried out by Mohammadi et al. in 2018 found that hesperidin supplementation did not have any significant effects on the serum lipid profile, which includes TC, TG, LDL, and HDL (64). The results of our systematic review contrast with those of the aforementioned studies, which may be attributed to differences in the number and heterogeneity of the studies included. Hesperidin has been shown to improve lipid metabolism by reducing serum levels of TG, TC, and LDL, as well as liver steatosis and adipose tissue. Furthermore, as obesity is closely linked to lipid metabolism, hesperidin also affects obesity. The mechanism by which hesperidin exerts its function involves various pathways, including the inhibition of cholesterol synthesis *via* the downregulation of retinol-binding protein (RBP), heart fatty acid-binding protein (H-FAB), and cutaneous fatty acid-binding protein (C-FAB) expression. This ultimately results in an improvement in hypercholesteremia and fatty liver in animal models, such as rats (34). Hesperidin also works by inhibiting the activity of two enzymes involved in cholesterol biosynthesis: 3-hydroxy-3-methylglutaryl-CoA (HMG-CoA) reductase and Acyl coenzyme A - cholesterol acyltransferase (ACAT). ACAT is responsible for converting intracellular cholesterol into cholesteryl ester in the rough endoplasmic reticulum of various tissues. In animal models, ACAT inhibitors have been shown to effectively reduce LDL levels (65, 66). HMG-CoA reductase is an enzyme that catalyzes the conversion of HMG-COA to mevalonate, which is a crucial step in cholesterol synthesis. Inhibitors of this enzyme have been demonstrated to effectively lower cholesterol levels in animal models and humans (67). A study demonstrated that hesperidin could inhibit HMG-COA reductase and ACAT, resulting in the reduction of TG and TC levels in mice (68). Hesperidin has also been shown to reduce serum TG in patients with hypertriglyceridemia. High levels of apo C-2 and apo E have been observed in individuals with hypertriglyceridemia. However, studies have shown that hesperidin administration can reduce the levels of these two factors. Apo C-2 and apo E are typically bound to VLDL, which is rich in TG. High concentrations of these two factors are associated with reduced VLDL catabolism. Patients with high TG often have high VLDL levels due to deficiencies in VLDL catabolism. Hesperidin administration has been found to enhance VLDL catabolism, which can reduce TG and LDL levels. Hesperidin may also activate lipoprotein lipase (LPL), an enzyme that hydrolyzes TG, further contributing to the reduction of TG levels (69). Hesperidin also reduces apolipoprotein B secretion in the human liver cell line, which is a principal component of LDL. This suggests that hesperidin may be involved in suppressing excess LDL secretion in the liver (70). Furthermore, hesperidin has been found to upregulate LDL receptors in human hepatoma cell lines, thereby increasing the uptake and degradation of LDL (71). These mechanisms, along with the inhibition of cholesterol biosynthesis and ACAT, and activation of LPL, could explain the effects of hesperidin on lipid profiles, including TC, TG, and LDL.

Our meta-analysis also revealed that the efficacy of hesperidin in reducing TG levels was influenced by certain factors. Subgroup analysis indicated that a longer intervention duration of more than six weeks and a daily dose of more than 500 mg were associated with a greater reduction in TG levels. Additionally, the supplementation of hesperidin was found to be more effective in reducing TG levels in obese subjects and participants younger than 50 years old. Our findings also suggest that hesperidin is more effective in reducing TG levels when the baseline TG of participants is higher than 150 mg/dL. The bioavailability of hesperidin in the blood of participants may be affected by their microbiome, as colon flora can convert hesperidin into insoluble metabolites that cannot be efficiently absorbed (13). Although hesperidin supplementation significantly reduced serum TC levels, subgroup analysis revealed it only reduced TC levels in studies with these specific characteristics: studies with obese participants (BMI > 30), baseline TC higher than 200, trial duration longer than six weeks, and intervention dose of more than 500 mg per day, or studies with participants younger than 50 years old. Similar results like TG were seen in TC in subgroup analysis. It is necessary to study what dosage and duration of hesperidin intervention are needed to reach a threshold of bioavailability in blood to affect TC in participants (72). The findings of this study suggest that hesperidin has a lowering effect on LDL levels. However, this effect was observed only in subgroups of participants who were obese (BMI > 30), younger than 50 years old, free from any history of cardiovascular disease, or had taken a high intervention dose (>500 mg/day) of hesperidin. As hesperidin has been shown to affect body weight in participants, it may exert a synergistic effect on LDL levels by modulating body weight. Notably, a higher intervention dose of hesperidin may increase its bioavailability, which may further enhance its LDL-lowering effects (72).

This meta-analysis showed supplementation of hesperidin had no effects on FBG and HOMA-IR when pooled effects sizes were included. A systematic review conducted by Tadros et al. in 2021 to evaluate the impact that hesperidin in 100% orange juice had on chronic disease biomarkers showed inconclusive results of its effect on insulin (62). In 2020, Shams-Rad et al. conducted a systematic review of the impact of hesperidin supplementation on blood glucose. The review found that hesperidin did not have a significant effect on fasting blood glucose, insulin, or homeostatic model assessment of insulin resistance (HOMA-IR). Moreover, there was no significant study heterogeneity observed, regardless of the study design or the health status of the participants. The subgroup analyses did not reveal any differences based on these factors. Overall, these findings suggest that hesperidin supplementation may not have a significant effect on blood glucose markers, based on the available evidence (73). Another systematic review conducted in 2019 evaluated the effects of hesperidin consumption on cardiovascular risk biomarkers in animal studies and human randomized clinical trials. Although hesperidin had beneficial effects in reducing glucose levels in animal models, no consensus was achieved considering hesperidin’s effect on cardiovascular risk biomarkers in humans. Glucose levels and insulin were evaluated in human studies, but no significant changes were found in any of them (63). We also did not find any significant effect on FBG or HOMA-IR; however, when we performed subgroup analyses, we found that hesperidin was effective in reducing FBG, particularly in cases where the dosage exceeded 500 mg/d and the intervention duration lasted more than six weeks. Furthermore, the positive effects of hesperidin were observed in subgroups of individuals with a baseline FBG level of 100 or higher, those with cardiovascular disease (CVD), and individuals younger than 50 years old. These findings suggest that hesperidin may be beneficial in managing FBG levels under certain conditions and in specific populations. It is possible that in studies where the hesperidin dose was lower than 500 mg per day or the duration was shorter than six weeks, the circulating concentrations of hesperidin may not have been sufficient to affect FBG levels in serum. Therefore, a higher dosage or longer duration of hesperidin supplementation may be required to observe significant effects on FBG levels. Further research is needed to determine the optimal dosage and duration of hesperidin supplementation needed to produce beneficial effects on FBG levels in serum (13).

Our analysis also revealed a non-significant dose–response effect of hesperidin dosage on the reduction of serum FBG levels, with a gradually decreasing trend observed from 300 to 1,000 mg of hesperidin per day. This suggests that hesperidin may exert its FBG-lowering effect through a wide range of dosages. We also found a significant U-shaped duration response of hesperidin supplement duration on FBG levels. While a 3- to 4-week intervention increased FBG levels, longer interventions of more than 6 weeks up to 12 weeks significantly reduced FBG levels. These findings suggest that hesperidin supplementation for more than 6 weeks may be the most effective in reducing FBG levels.

In addition, our non-linear dose–response analysis revealed a significant association between hesperidin supplementation dose and insulin levels. There was a significant dose–response effect of hesperidin on insulin levels, with insulin levels gradually decreasing with hesperidin in the range of 300 mg/day to 1,000 mg/day. Moreover, 1,000 mg/day of hesperidin was found to be the most effective in lowering insulin levels. The duration-response analysis also found a significant U-shaped duration-response effect of hesperidin on insulin levels. Insulin levels showed an increasing trend when the duration of intervention was between 3 to 8 weeks, while a decreasing trend was seen when the duration of intervention was more than 8 weeks. These findings suggest that longer interventions of more than 8 weeks may be needed to observe significant reductions in insulin levels with hesperidin supplementation.

The antidiabetic effects of hesperidin have been demonstrated in animal models, with hesperidin exerting its effects by stimulating insulin secretion, stimulating glucose uptake in peripheral tissue, inhibiting gluconeogenesis (downregulation of glucose-6-phosphatase), and activating glycolysis (upregulation of glucokinase) (74). Hesperidin could also affect insulin levels and insulin resistance by modulating inflammation since it has been shown that inflammatory markers, including leptin, IL-6, and TNF-α, play a role in the pathogenesis of DM and the development of insulin resistance. Although all shreds of evidence in animal models point to the antidiabetic effect of hesperidin, its effect on glucose metabolism in humans is inconclusive and hence needs further studies to be elucidated (13).

This meta-analysis indicated that hesperidin supplementation significantly lowered the TNF-α level in serum. A systematic review conducted in 2021 that evaluated the impact hesperidin in 100% orange juice had on chronic disease biomarkers reported that hesperidin in orange juice decreased IL-6 and TNF-α compared to the control group (62). Reduction in IL-6 and TNF-α in that systematic review is in line with ours. We also found a reduction in IL-6 and TNF-α after hesperidin supplementation; however, a reduction in IL-6 was only seen in some subgroups. A systematic review conducted by Lorzadeh et al. in 2019, which included only six studies, reported that hesperidin supplementation significantly reduced vascular cell adhesion molecule 1 (VCAM-1), although it had no significant effect on IL-6 or CRP. However, hesperidin reduced CRP levels in studies in which the intervention duration was longer than 4 weeks (75). Our findings are consistent with this previous systematic review in that we also did not find any significant effect on either IL-6 or CRP. However, we did observe a significant reduction in IL-6 levels when the hesperidin dosage was greater than 500 mg per day.

This meta-analysis indicated hesperidin supplementation significantly lowered the TNF-α level in serum. However, it did not affect the TNF-α level when a low dose (<500) was used and in studies conducted on men only or normal-weight participants. A low dose of hesperidin supplementation may not be sufficient to reduce TNF-α levels due to its low bioavailability (72). It should be noted that only one study included male participants in the subgroup analysis, and therefore, the reliability of these results may be limited.

Hesperidin, as a flavonoid, seems to have anti-inflammatory properties. Inflammation is a complex process that includes many inflammatory mediators such as iNOS (inducible nitric oxide synthase) and cyclooxygenase-2 (COX-2), which enhance the nitric oxide level and prostaglandins. Several cytokines are also related to the inflammatory process and are elevated in these processes, including TNF-α, IL-1β, and IL-6. These mediators and cytokines are regulated by transcription factors, of which kappa-light-chain-enhancer of activated B cells (NF-κB) has the central role. NF-κB signaling pathway also activates mitogen-activated protein kinases (MAPKs), which are involved in inflammatory processes (76). Many studies have investigated the effect of hesperidin on inflammation in cellular and animal models. They have demonstrated that hesperidin could alleviate inflammation by suppressing the expression of iNOS and COX-2 and hence decreasing the prostaglandin level. Hesperidin also decreases TNF-α, IL-1β, IL-6, and MAPKs levels. Through these mechanisms, hesperidin could exert anti-inflammatory effects (76–80).

This meta-analysis indicated that hesperidin supplementation did not affect BMI and WC but an increasing effect was detected on weight. In a systematic review conducted by Pla-Paga et al. in 2019, hesperidin did not affect weight or BMI (63). Our results are not in line with the previous systematic review. This could be due to different studies and populations and their mean weight that were included in our systematic review.

Our meta-analysis revealed that hesperidin induced weight gain in participants, specifically in those with intervention durations of less than 6 weeks, in those with intervention doses of more than 500 mg per day, in participants older than 50 years old, and in participants with a CVD history. Increasing intervention dose and duration improved hesperidin effects probably by increasing its bioavailability in the blood (13). However, there seemed to be some justification for several transcription factors, including CCAAT/enhancer binding protein (C/EBPs) and peroxisome proliferator-activated receptor gamma (PPAR-γ), promoting lipogenesis during the development of adipocytes. Citrus flavonoid extracts prevent intracellular triglyceride and fat formation and decrease PPAR-243 expression. Citrus flavonoids are thought to be the primary factor reducing lipid accumulation in HepG 2 cells by inhibiting oleic acid-induced production of miR-122 and miR-33 and their target messenger ribonucleic acids (mRNAs), fatty acid synthase (FAS) and carnitine palmitoyl transferase 1 (CPT1) (81, 82). Flavonoids such as hesperidin significantly lower the triacylglycerol concentration of preadipocytes, and it has been noted that the chemical structure of flavanones is the most efficient at suppressing adipogenesis (83, 84). At first sight, there might be controversy with our weight gain result but absolute body weight consists of fat, fat-free mass (FFM), muscle mass, and others, and, therefore, it is likely that hesperidin could decrease fat mass but increase muscle mass or one of the other components of absolute body weight. In addition, one probable mechanism includes hesperidin promoting nuclear localization of nuclear protein MyoD. MyoD plays an important role in the modulation of myogenic precursors, induces myoblast differentiation, and is expressed exclusively in skeletal muscle mass. The interaction of MyoD with target gene promoters and transcription factor enhances MyoD mediated myogenic gene transcription and myogenic differentiation, which induce hypertrophy of skeletal muscle mass. This mechanism could probably be responsible for anthropometric changes (85–87).

Our findings indicated that hesperidin supplementation decreased SBP in overall effect; however, did not affect DBP. Pla-Paga et al. conducted a systematic review in 2019 to evaluate the effects of hesperidin consumption on cardiovascular risk biomarkers in animal studies and human randomized clinical trials. Body weight, BMI, body fat, SBP, DBP, glucose level, insulin, TC, LDL, HDL, and TG were evaluated in human studies, but no significant changes were found in any of them (63). In addition, another systematic review and meta-analysis conducted by Mohammadi et al. in 2018 revealed that hesperidin supplementation had no effects on SBP or DBP (64). We found that hesperidin can decrease SBP, however, it does not affect DBP. Controversial results regarding the effect of hesperidin on blood pressure could be due to different studies and the participants with different health statuses that were included in the different systematic reviews.

Subgroup analysis showed that hesperidin supplementation is more effective in reducing SBP when the duration of the intervention is shorter than six weeks or when participants are overweight (BMI:25–29.9). Although it is not clear why a shorter intervention duration is more effective in reducing SBP, it could be due to the compensating mechanisms that activate in the long term and reduce the effect of hesperidin. Furthermore, different hesperidin metabolites, which have various extents of hypertensive effects, should be considered when interpreting research results (35).

Mechanistic evidence of the effect of hesperidin on CVD risk factors has been investigated extensively in animal models but to less extent in human subjects; however, its mechanism of action is not entirely discovered. Hesperidin possesses anti-inflammatory and anti-oxidative activities and also affects lipid and glucose metabolism. Understanding the effects of hesperidin and its mechanism of action is of great importance for using this substance as a treatment for various diseases. The anti-hyperglycemic effect of hesperidin has been investigated in mice. Hesperidin exerts its effects on FBG by modulating the activity of key enzymes in glucose metabolism. Hesperidin maintains glucose metabolism by regulating PPAR-c, a nuclear transcription factor (88). Hesperidin is a key enzyme of glucose catabolism and induces glucokinase expression, which is involved in sensing glucose levels in the body. Hesperidin also inhibits gluconeogenesis by decreasing the level of glucose-6-phosphatase (89) and has been shown to have antidiabetic effects in streptozotocin-induced type 2 diabetic rats. Hesperidin exerts its effects by stimulating insulin secretion, stimulating glucose uptake in peripheral tissues, activating gluconeogenesis, and inhibiting glycogenolysis (74). Although the hesperidin effect on glucose metabolism in mice has been investigated extensively, its effects in humans are not as clear as in mice. Some studies have been conducted to explain the mechanism by which hesperidin reduces blood pressure. The blood pressure-lowering effect of hesperidin could be due to its role in inducing the production of nitric oxide (NO) by vascular endothelium and inhibiting nicotinamide adenine dinucleotide phosphate (NADPH) oxidase activity. NO causes vasodilation and hence lowers blood pressure by relaxing smooth muscles in blood vessels (90). Flavonoid-rich food inhibits the angiotensin-converting enzyme, hence reducing the angiotensin 2 level, which is the active form of angiotensin with vasoconstrictor activity. Hesperidin as a flavonoid may also lower blood pressure through this mechanism (91). Hesperidin has been shown to inhibit inflammatory responses. Our results align with two *in vitro* studies that showed that hesperidin inhibits mast cell inflammatory responses and inflammatory cytokine secretion and decreases TNF-α activity (92, 93). Hesperidin inhibits NF-κB, and since NF-κB induces the expression of pro-inflammatory factors (including TNF-α), this can explain the anti-inflammatory effects of hesperidin (94). Hesperidin inhibits NF-κB activation and IL-6 production by increasing adiponectin. Adiponectin induces PPAR-γ activation, which inhibits NF-κB activation and IL-6 production (95). Dose-dependent anti-inflammatory effects of hesperidin have been reported in mice (96). Although several studies have demonstrated the hypoglycemic, lipid-lowering, and anti-inflammatory activities of hesperidin in animal models and human cell lines, further clinical trials and mechanistic studies are needed to validate the therapeutic effects of hesperidin in humans.

This meta-analysis has some limitations and strengths that should be addressed. Participants in different studies had varying degrees of health statuses, different mean ages, and different BMIs, which affected the heterogeneity of the data. Different study designs could lead to heterogeneity in results and should be considered seriously. Heterogeneity in the included population, hesperidin dosage, and intervention duration was seen in studies. Hesperidin can be found in consumed food and beverages, but its bioavailability is affected by the food matrix in which it is consumed, so controlling the diet of participants is of great importance. However, not all studies vigorously monitored dietary intake (97). Furthermore, various companies produced the hesperidin supplements used in the different studies, which could affect the bioavailability of this compound in participants’ blood. Interindividual varieties, for instance, microbiome variation in different participants could also have affected the bioavailability of hesperidin (98). Since studies had not measured the bioavailability of this compound, the association between cardiovascular risk factors and the exact concentration of hesperidin in the blood could not be evaluated. Different laboratory kits were used to measure CVD biomarkers in different studies. Intra-assay and inter-assay variation could affect the results and lead to bias in the interpretation of the results. Side effects were not found due to hesperidin supplementation in most studies. Almost all systematic reviews that we included had a low risk of bias according to the Cochrane criteria; however, Egger’s test indicated evidence of bias in the meta-analysis for the effect of hesperidin supplementation on TG, HDL, weight, and TNF-α. This could interfere with interpreting the results relating to TG, HDL, weight, and TNF-α. Due to all these limitations, more large-scale and rigorously controlled clinical trials are needed before hesperidin can be used as a human therapeutic. This is a comprehensive systematic review that included all RCTs, and no limitations were set in terms of date. We involved many CVD risk biomarkers to have a comprehensive systematic review regarding the effect of hesperidin on CVD risk factors. As the studies in this meta-analysis were included based on the inclusion criteria, we were able to perform subgroup analyses. The standardized methodology used in this systematic review and meta-analysis is one of its important strengths. Since heterogeneity was apparent among studies, we tried to understand the true effect of hesperidin by reducing heterogeneity using subgroup analysis. We used different statistical methods to assess the effect of hesperidin on CVD risk factors. The studies included in this meta-analysis were from five different countries, including European and Asian countries, which increases the generalizability of results.

## 5. Conclusion

This meta-analysis indicated that hesperidin supplementation had a lowering effect on TG, TC, and LDL serum levels, and it also lowered TNF-α and blood pressure. However, further well-designed RCTs and mechanistic studies are needed to elucidate the effect of hesperidin on CVD risk factors, specially FBG, insulin resistance, blood pressure, HDL, and inflammatory markers. It seemed that the effective dosage and duration of hesperidin supplementation for decrement of insulin level are approximately 1,000 mg/d and more than 8 weeks. In addition, the findings revealed a non-linear association between the duration of hesperidin intervention and FBG, with a decrease in FBG levels appearing after 6 weeks of hesperidin consumption.

## Data availability statement

The original contributions presented in the study are included in the article/Supplementary material, further inquiries can be directed to the corresponding authors.

## Author contributions

AK designed the study. AK and OA developed the search strategy, extracted the data, conducted the analyses, and assessed the risk of bias of the meta-analyses. NR, SF, and FG drafted the manuscript. FS, OA, and AK interpreted the results. FS, SK, and OA revised the manuscript. All authors read and approved the final manuscript.

## Conflict of interest

The authors declare that the research was conducted in the absence of any commercial or financial relationships that could be construed as a potential conflict of interest.

## Publisher’s note

All claims expressed in this article are solely those of the authors and do not necessarily represent those of their affiliated organizations, or those of the publisher, the editors and the reviewers. Any product that may be evaluated in this article, or claim that may be made by its manufacturer, is not guaranteed or endorsed by the publisher.

## Glossary

ACAT: Acyl cholesterol acyltransferase
C-FAB: Cutaneous fatty acid-binding protein
CVDs: Cardiovascular diseases
CPT1: Carnitine palmitoyl transferase
C/EBPs: CCAAT/enhancer binding protein
FAS: Fatty acid synthase
FFM: Fat-free mass
H-FAB: Heart fatty acid-binding protein
PPAR-γ: Peroxisome proliferator-activated receptor gamma
I square: I^2^
RBP: Retinol binding protein
RCTs: Randomized controlled trials
SEM: Standard Error of Mean
TC: Total cholesterol
TG: Triglyceride
LDL: Low-density lipoprotein
HDL: High-density lipoprotein
FBG: Fasting blood glucose
HOMA-IR: Homeostatic Model Assessment for Insulin Resistance
SBP: Systolic blood pressure
DBP: Diastolic blood pressure
CRP: C-reactive protein
IL-6: Interleukin 6
TNF-α: Tumor necrosis factor
BMI: Body mass index
WC: Waist circumference
SDs: Standard deviations
mRNAs: Messenger ribonucleic acids
NADPH: Nicotinamide adenine dinucleotide phosphate
PICO: Participant, Intervention, Comparison/Control, Outcome
GRADE: Grading of Recommendations Assessment, Development, and Evaluation
RBP: Retinol binding protein
H-FAB: Heart fatty acid-binding protein
C-FAB: Cutaneous fatty acid-binding protein
HMG-CoA: 3-hydroxy-3-methylglutaryl-CoA
VCAM-1: Vascular cell adhesion molecule 1
WMD: Weighted mean difference.
